# Supplementary material for: High-Throughput Sequencing to Identify Mutations Associated with Retinal Dystrophies
Source: Genes (Basel). 2021 Aug 20;12(8):1269. doi: 10.3390/genes12081269 (PMC8391535; doi:10.3390/genes12081269)

Supplementary Materials

Figure S1. Sanger sequencing and WES results of the patients in this study. f: forward; r: reverse.

Patient 001

Disease-associated gene: NR2E3

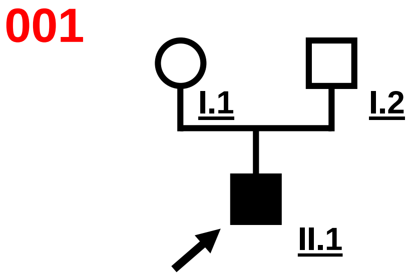

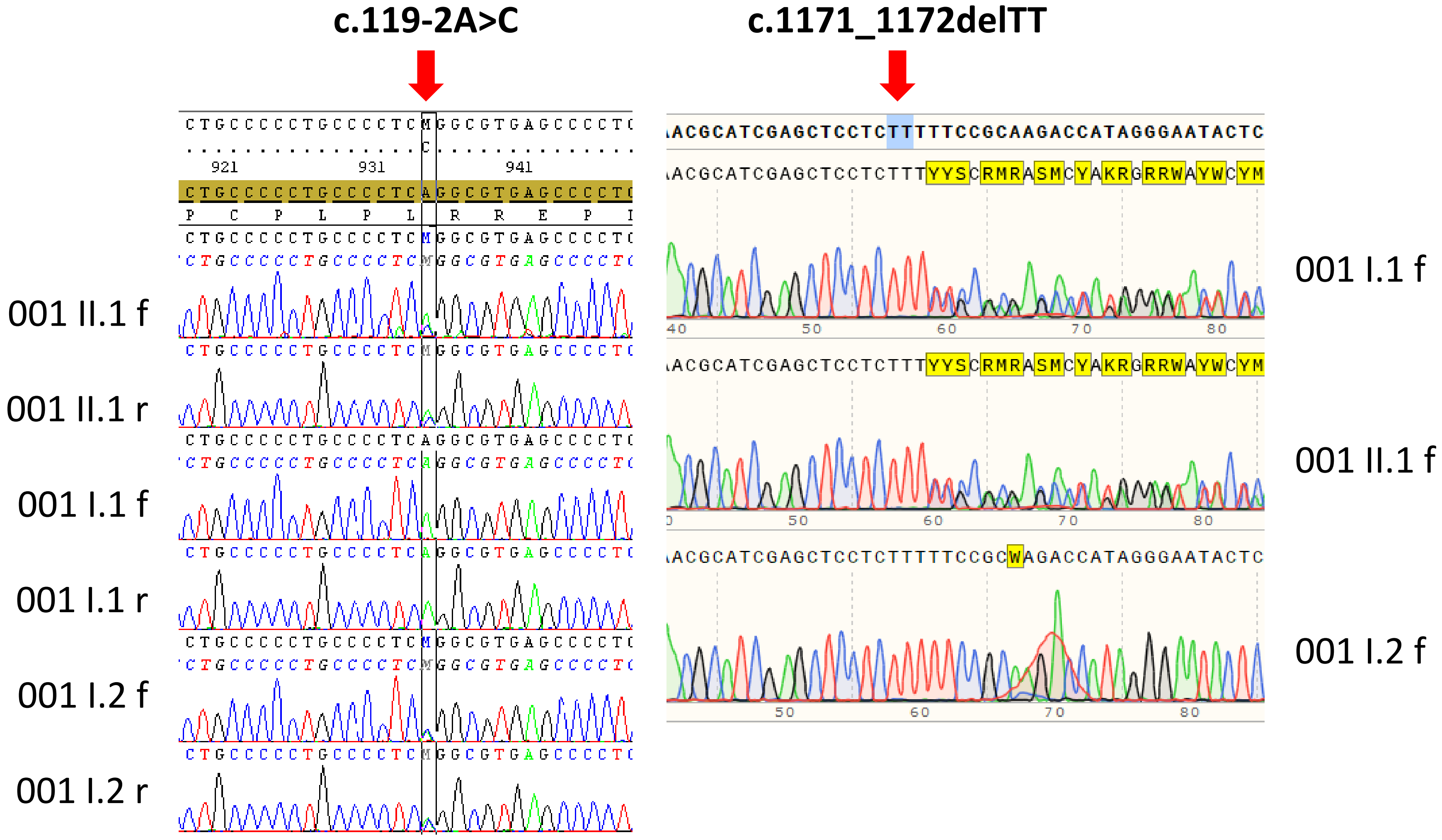

Patient 002

Disease-associated gene: USH2A

c.4714C>T (173x Total Count)

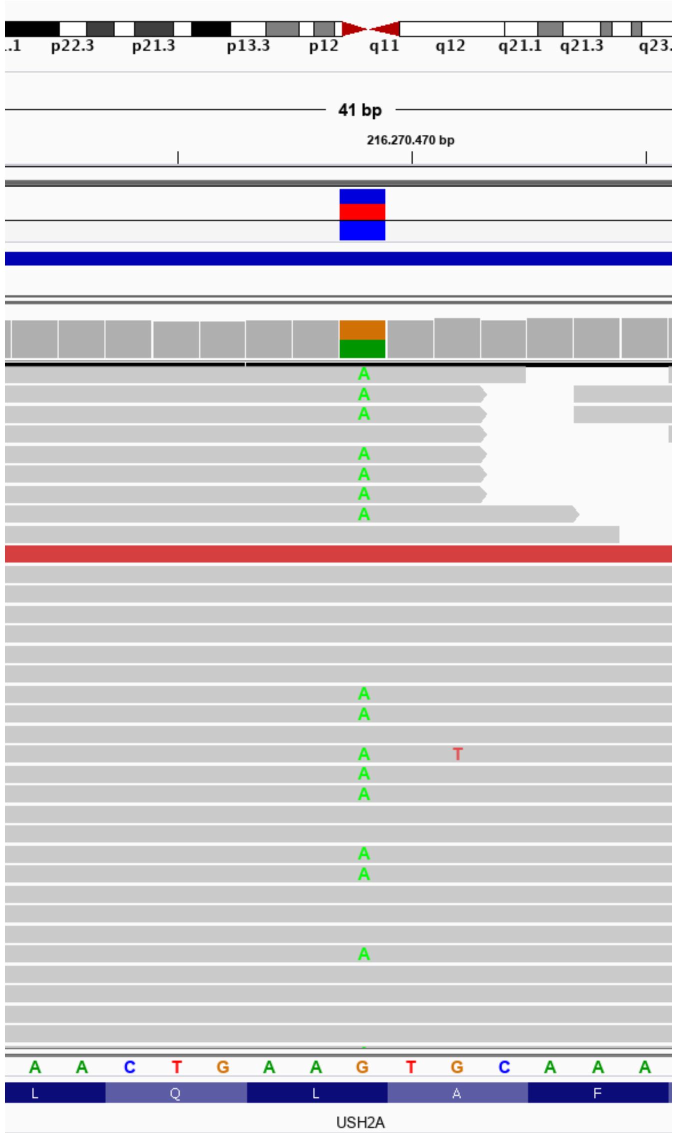

c.2299delG (63x Total Count)

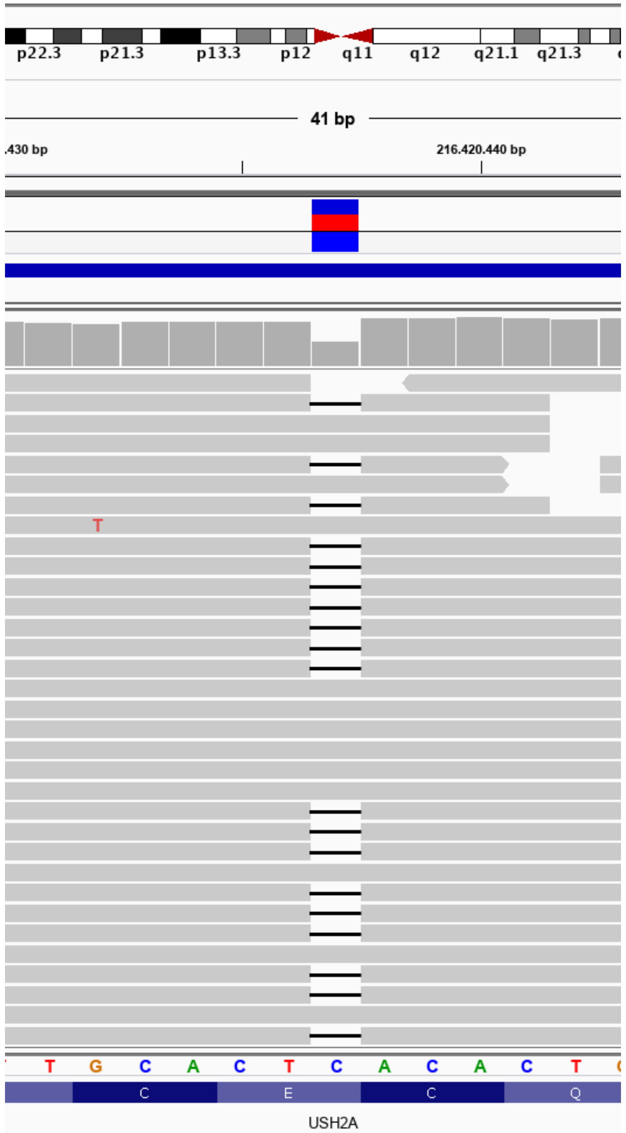

Chromosome 1

Chromosomal Position

NGS Reads

Patient 003

Disease-associated gene: USH2A

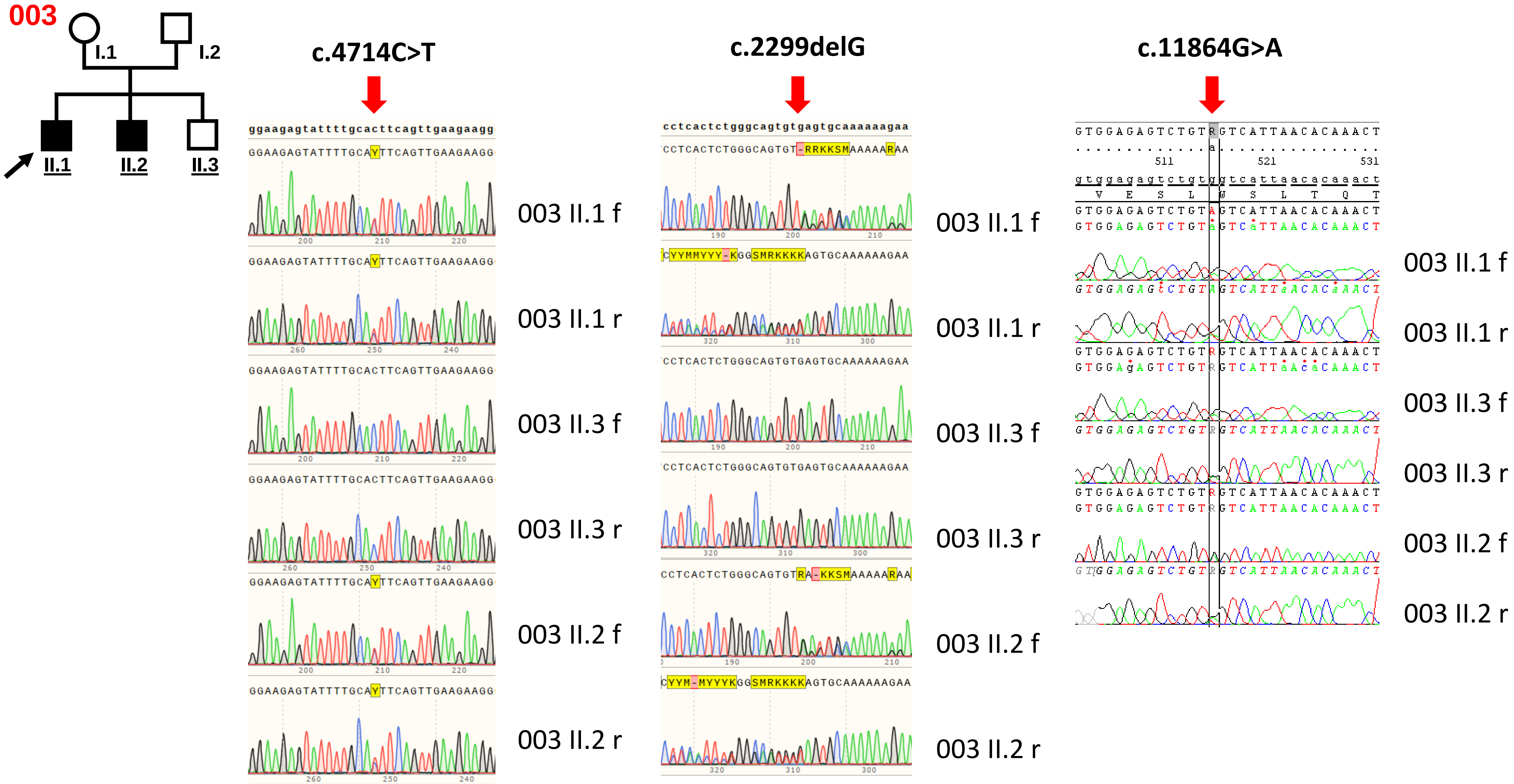

Patient 004

Disease-associated gene: USH2A

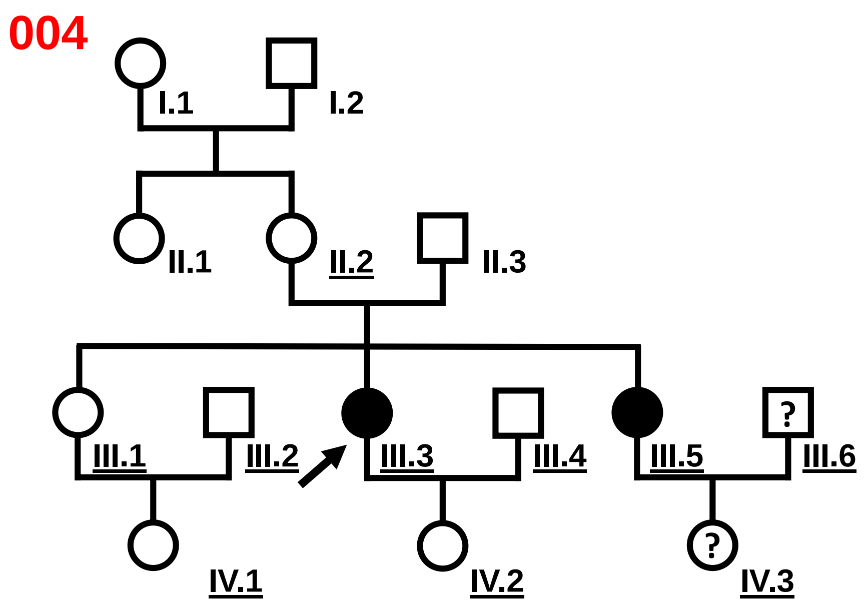

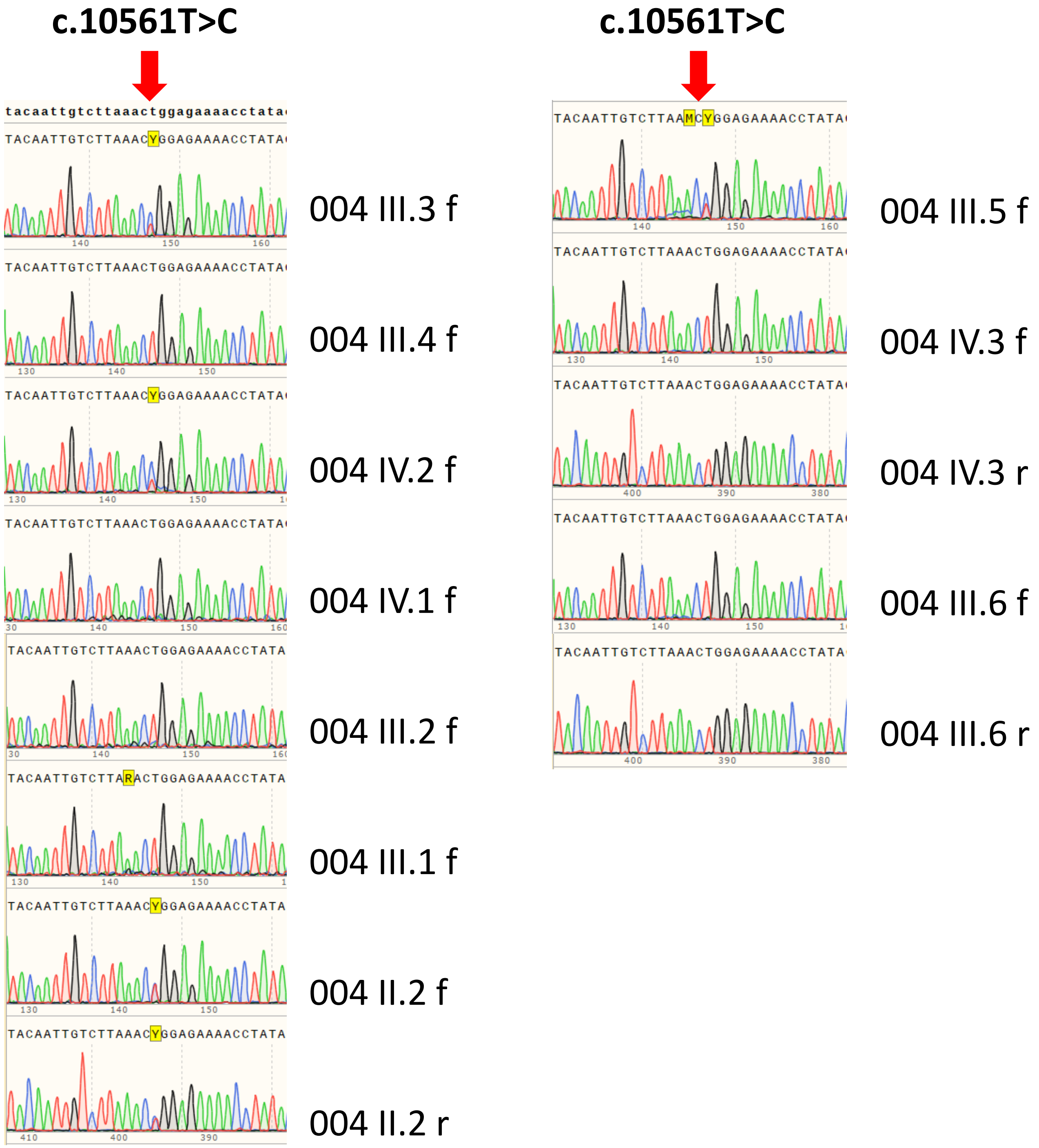

Patient 004

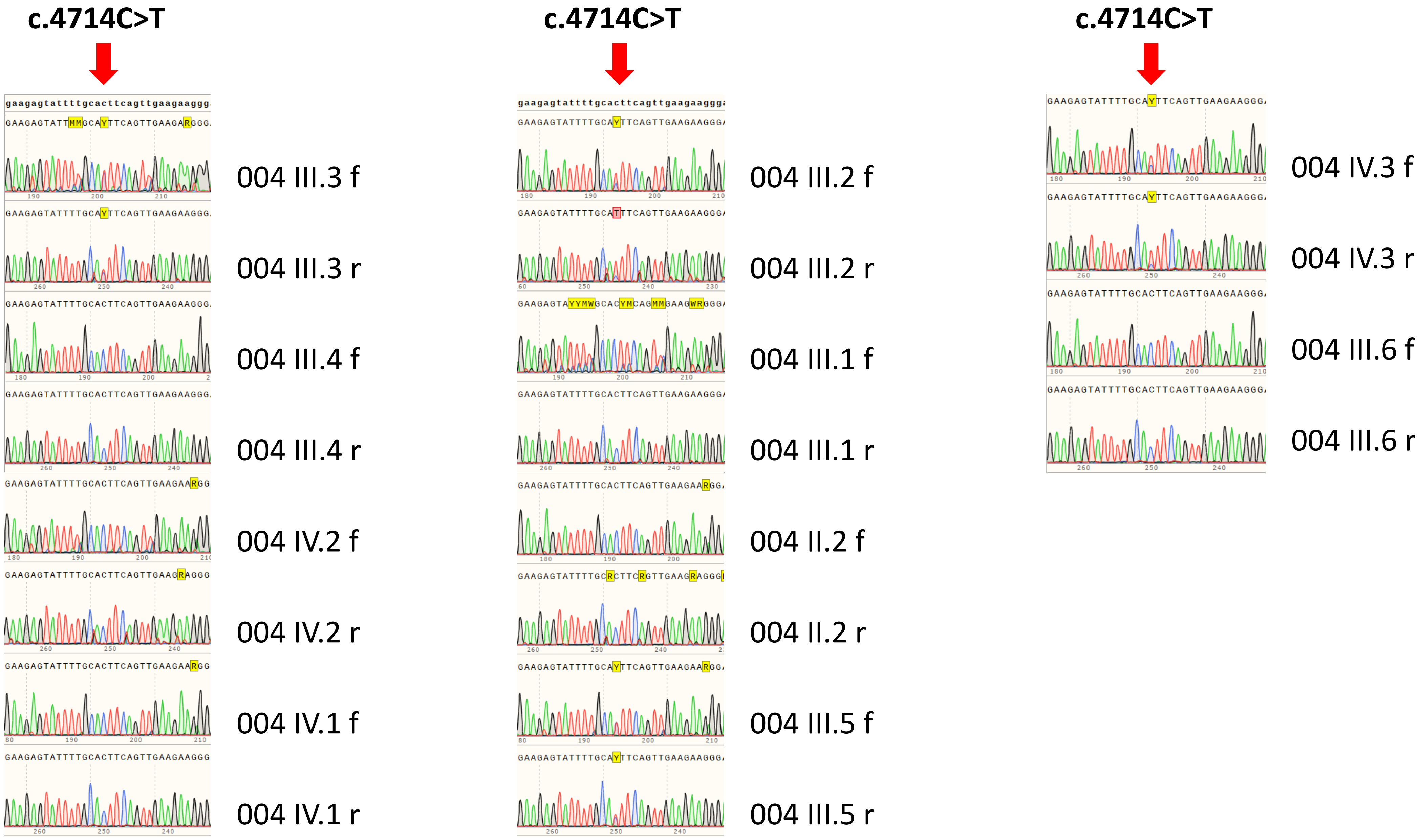
c.4714C>T
c.4714C>T
c.4714C>T
004 III.3 f
004 III.3 r
004 III.4 f
004 III.4 r
004 IV.2 f
004 IV.2 r
004 IV.1 f
004 IV.1 r
004 III.2 f
004 III.2 r
004 III.1 f
004 III.1 r
004 II.2 f
004 II.2 r
004 III.5 f
004 III.5 r
004 IV.3 f
004 IV.3 r
004 III.6 f
004 III.6 r

Patient 004

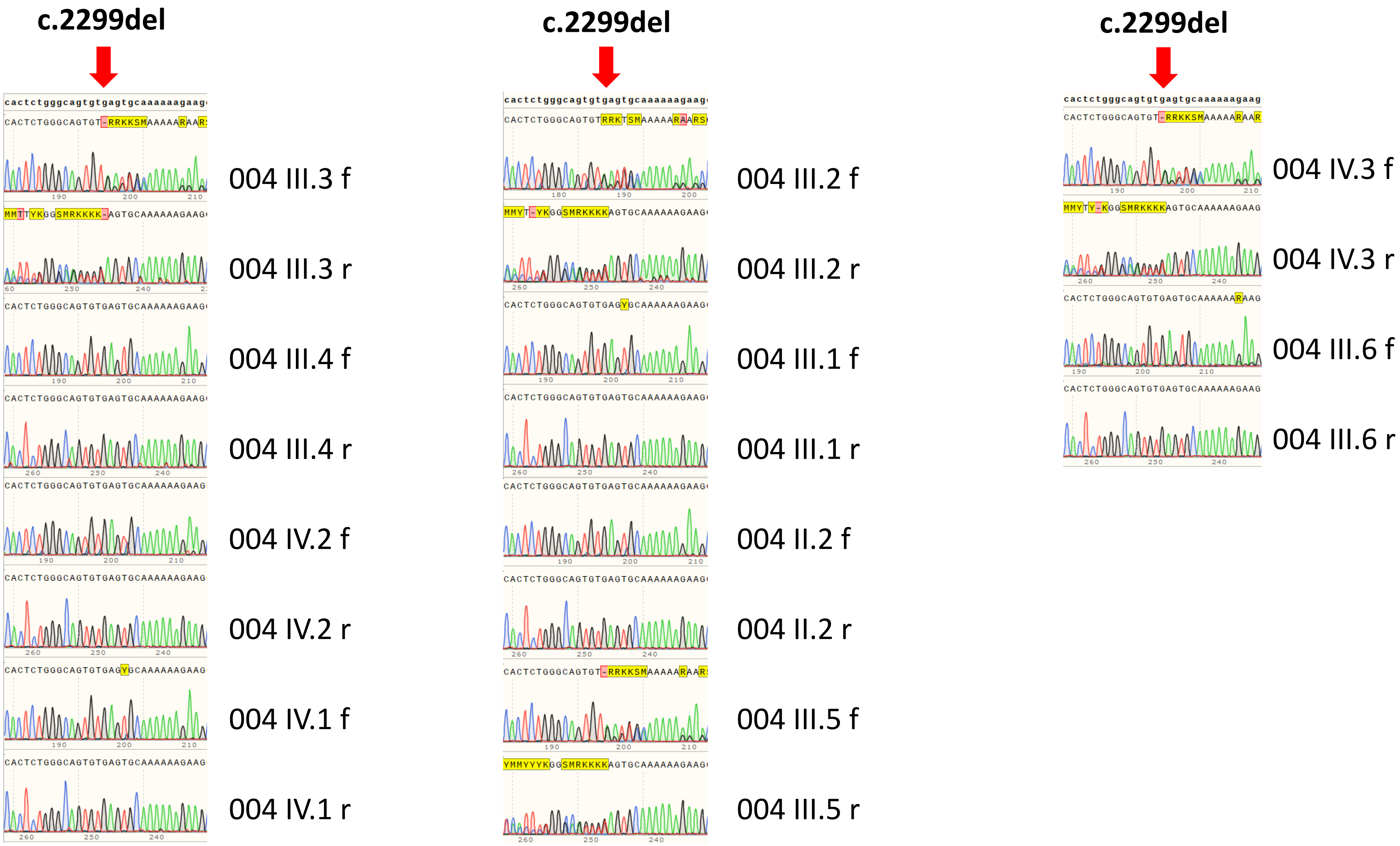
c.2299del
c.2299del
c.2299del
004 III.3 f
004 III.3 r
004 III.4 f
004 III.4 r
004 IV.2 f
004 IV.2 r
004 IV.1 f
004 IV.1 r
004 III.2 f
004 III.2 r
004 III.1 f
004 III.1 r
004 II.2 f
004 II.2 r
004 III.5 f
004 III.5 r
004 IV.3 f
004 IV.3 r
004 III.6 f
004 III.6 r

Patient 005

Disease-associated gene: c2orf71

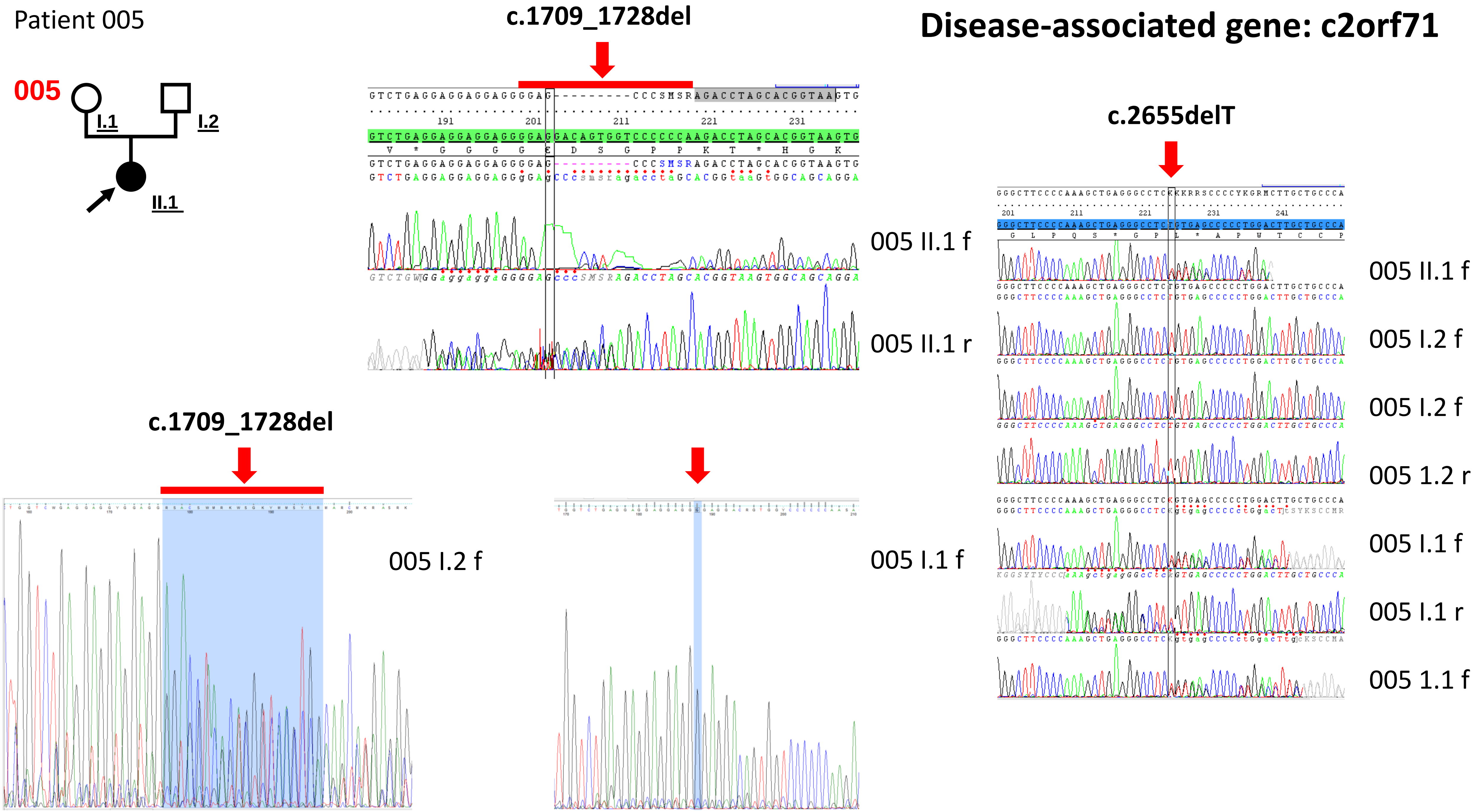

Patient 006

Disease-associated gene: RP1L1

c.3514C>A (85x Total Count) | c.130C>G (53x Total Count)

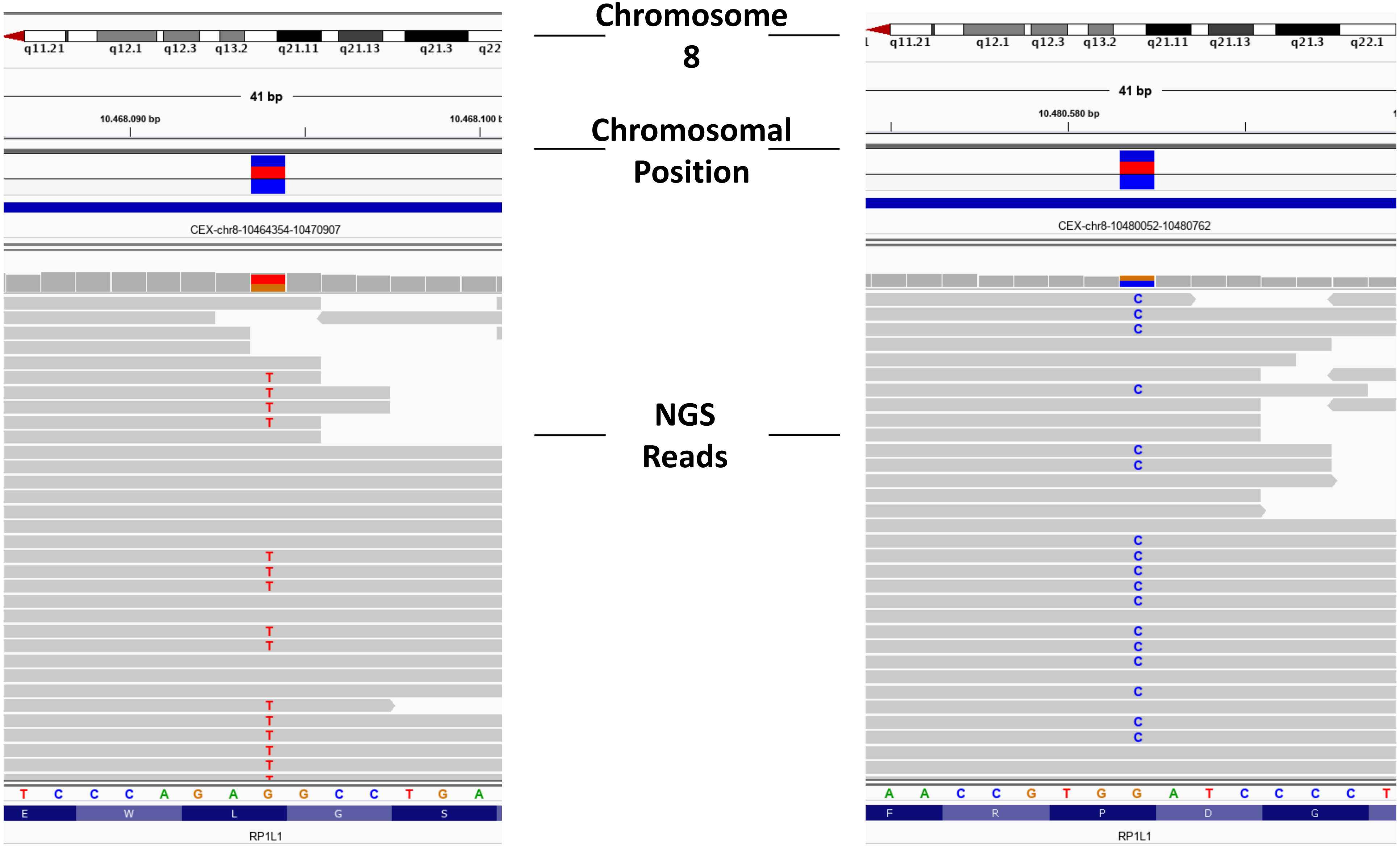

Patient 007

Disease-associated gene: RP1L1

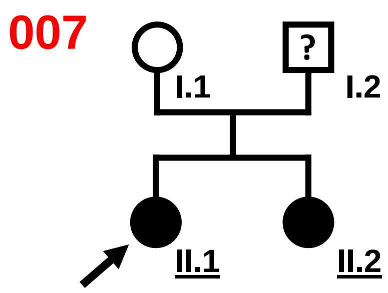

c.133C>T

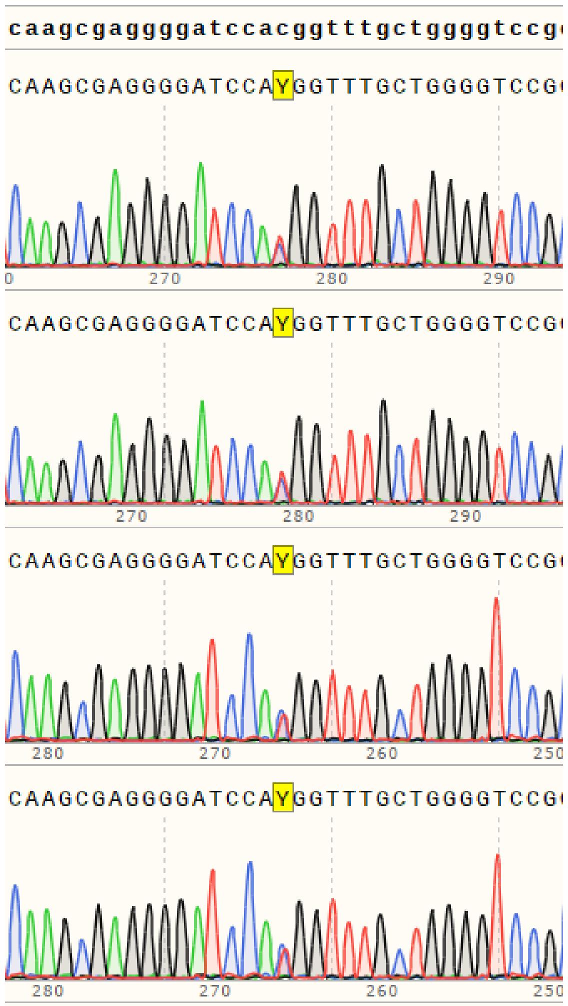

007 II.1 f

007 II.1 f

007 II.1 r

007 II.1 r

c.133C>T

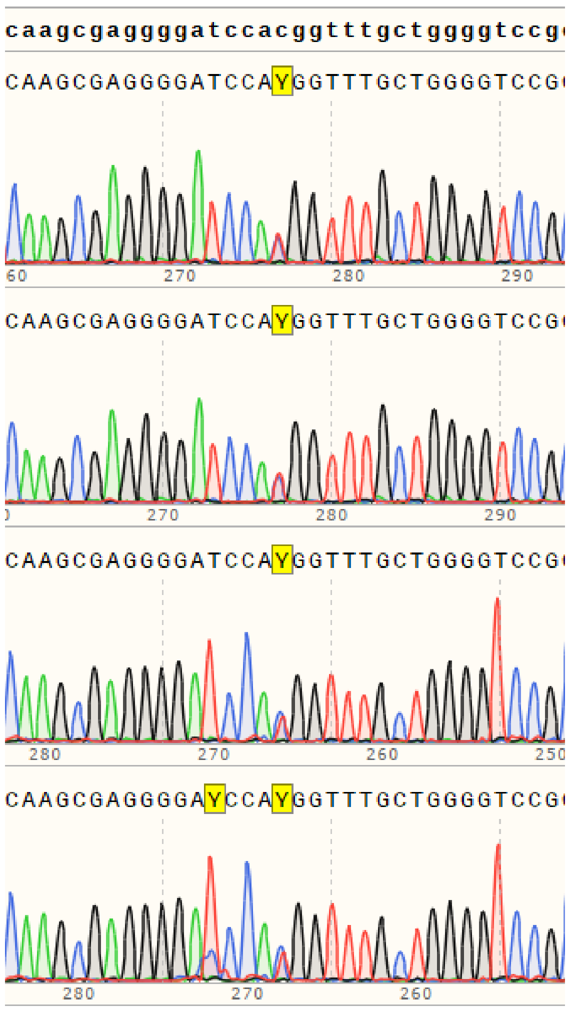

007 II.2 f

007 II.2 f

007 II.2 r

007 II.2 r

Patient 008

Disease-associated gene: RIMS1

c.1919C>A (26x Total Count)

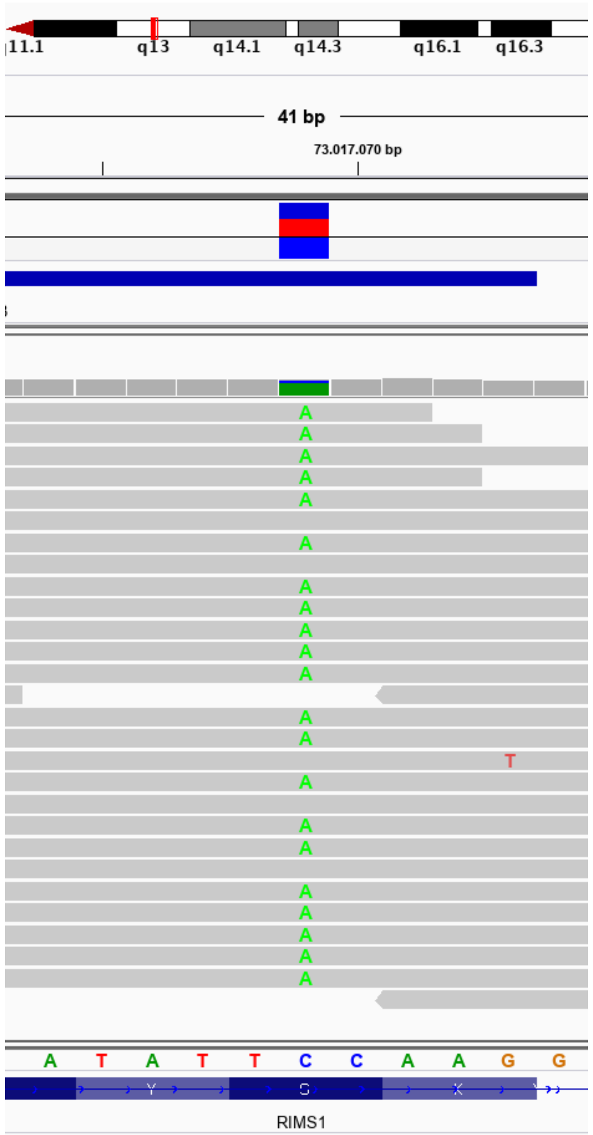

Chromosome 6

Chromosomal Position

NGS Reads

c.1919C>A

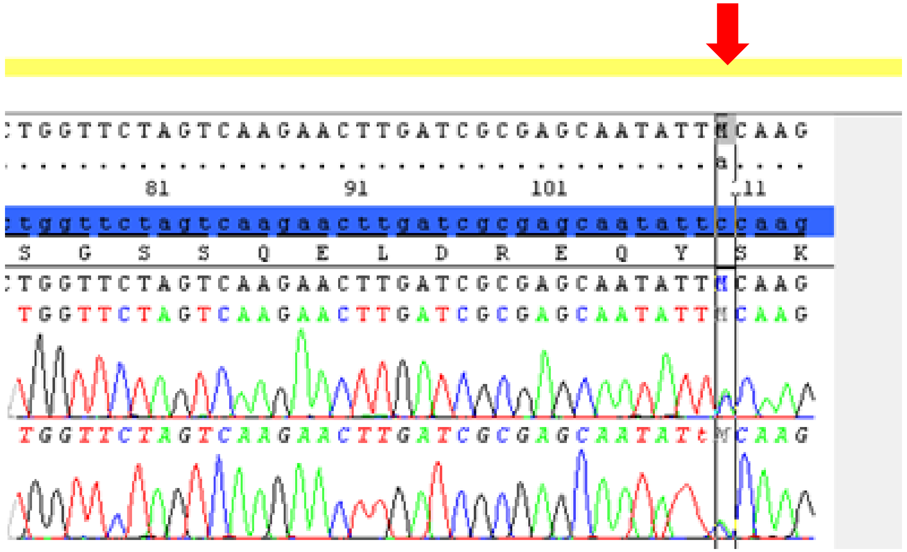

008 f

008 r

Patient 009

Disease-associated gene: PDE6A

c.607T>A (111x Total Count)

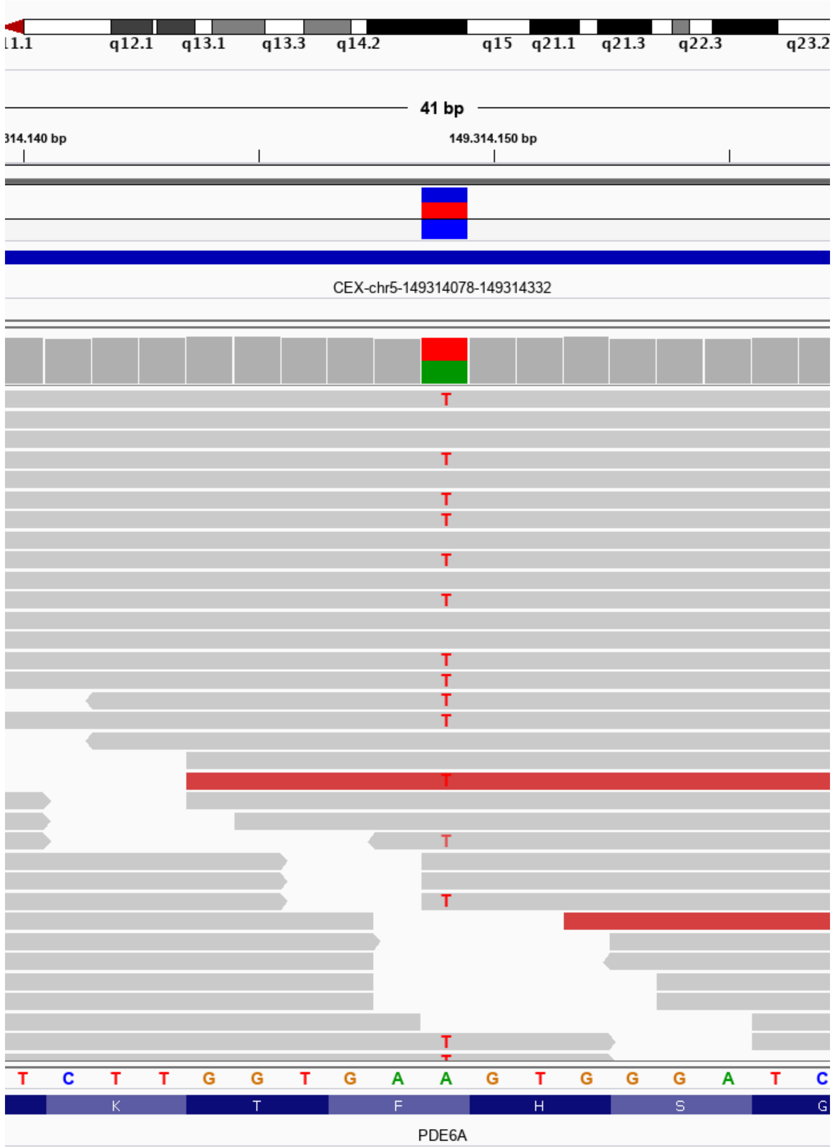

Chromosome 5

Chromosomal Position

NGS Reads

c.607T>A

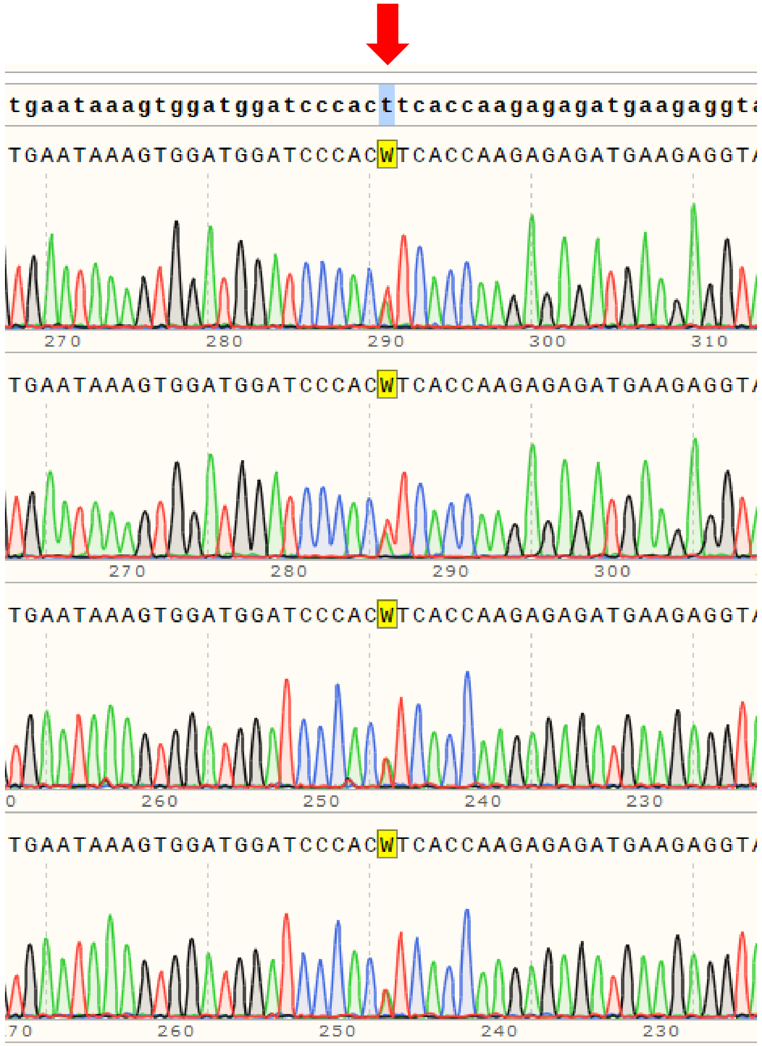

009 f

009 f

009 r

009 r

Patient 010

Disease-associated gene: RP1

c.5957G>A (96x Total Count)

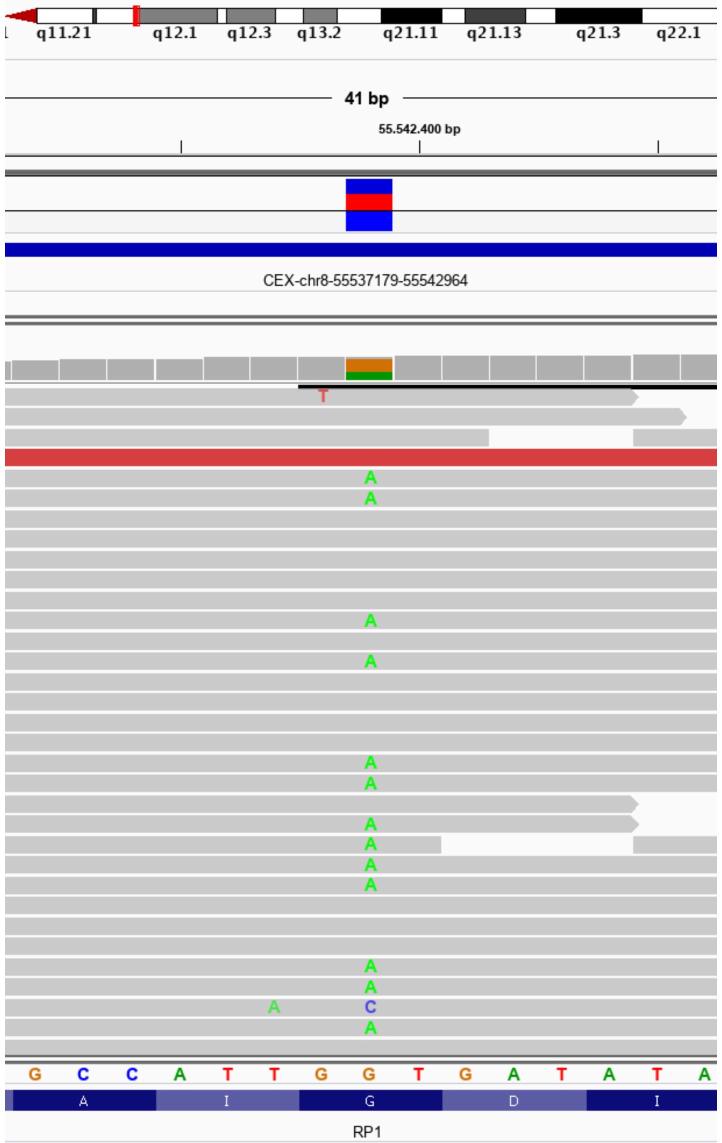

Chromosome 8

Chromosomal Position

NGS Reads

c.5957G>A

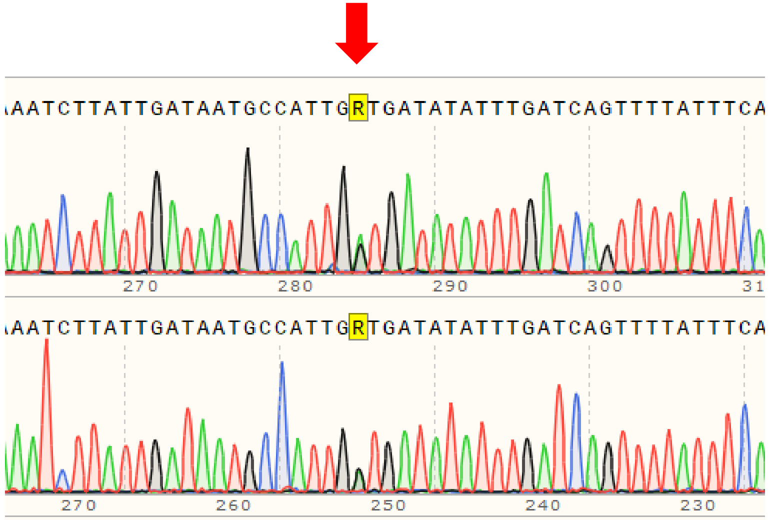

010 f

010 r

Patient 014

Disease-associated gene: PROM1

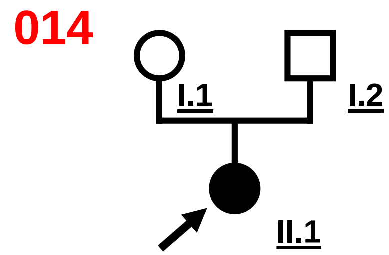

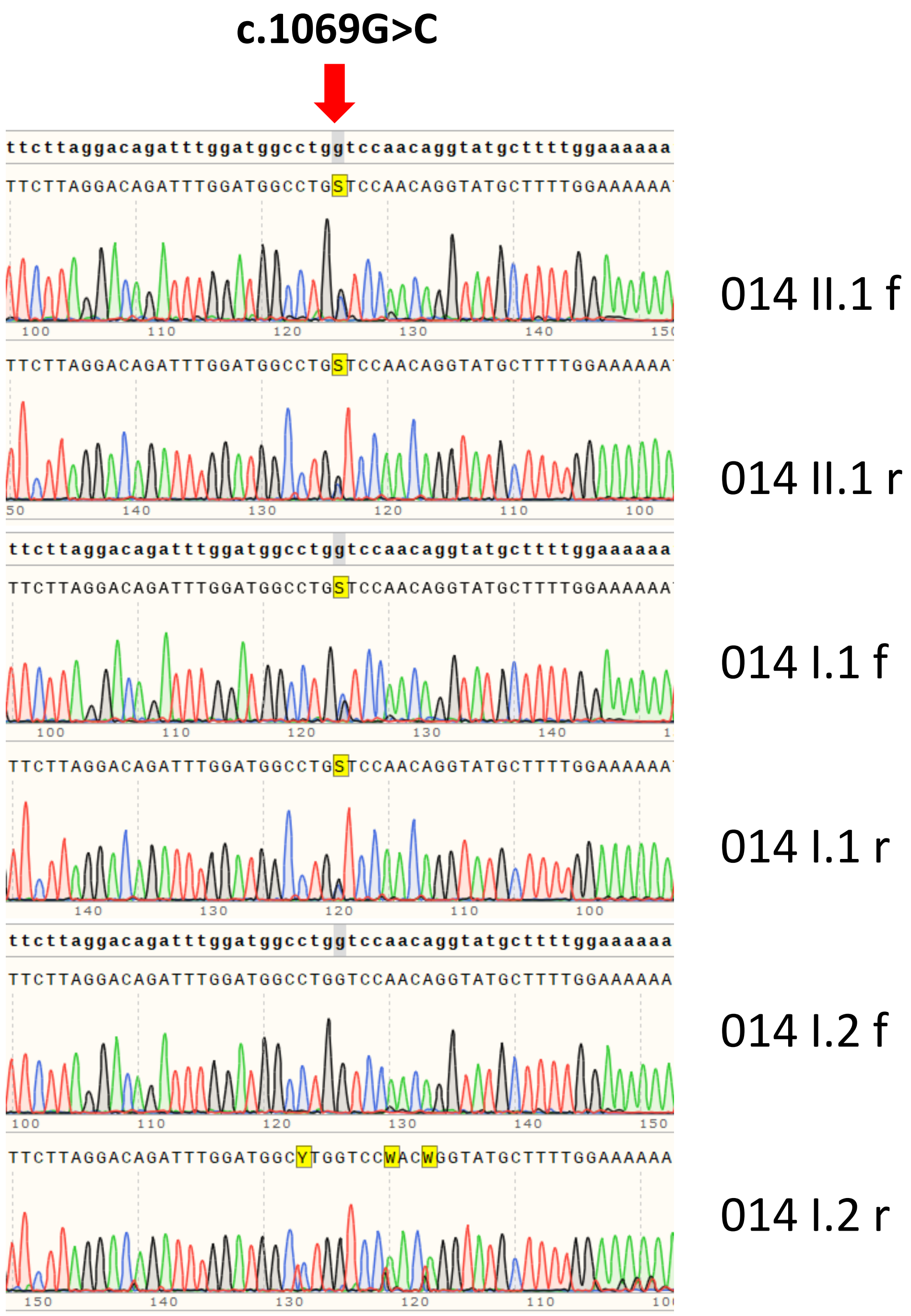

Patient 016

Disease-associated gene: SNRNP200

c.3260C>T (96x Total Count)

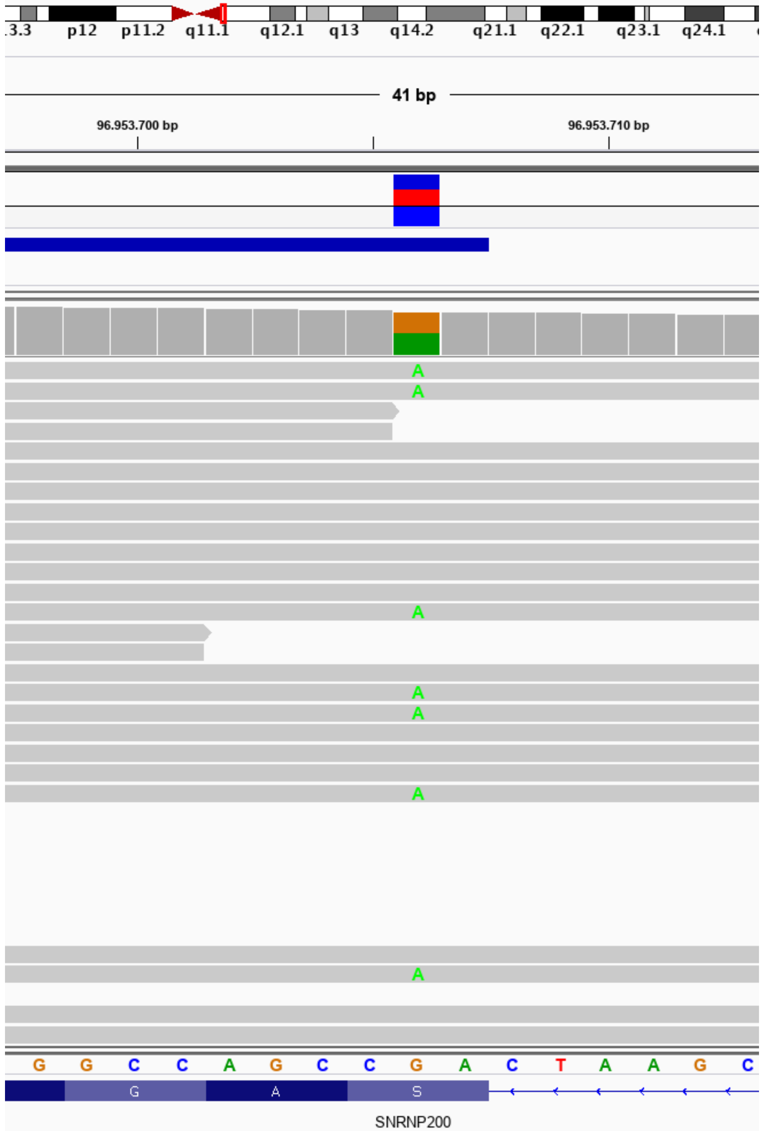

Chromosome 2

Chromosomal Position

NGS Reads

Patient 017

Disease-associated gene: RPE65

c.1154C>T (152x Total Count)

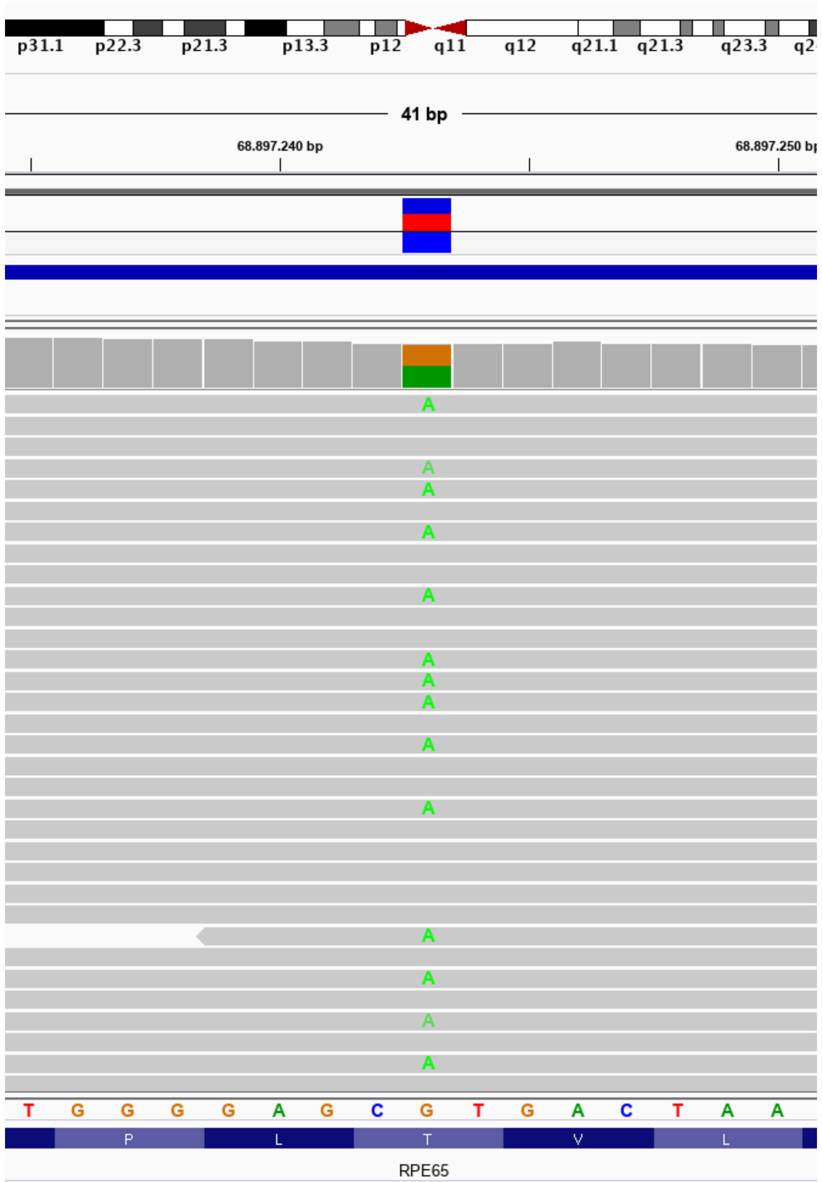

Chromosome 1

Chromosomal Position

NGS Reads

Patient 018

Disease-associated gene:PRPF8

c.1098+6del (48x Total Count)

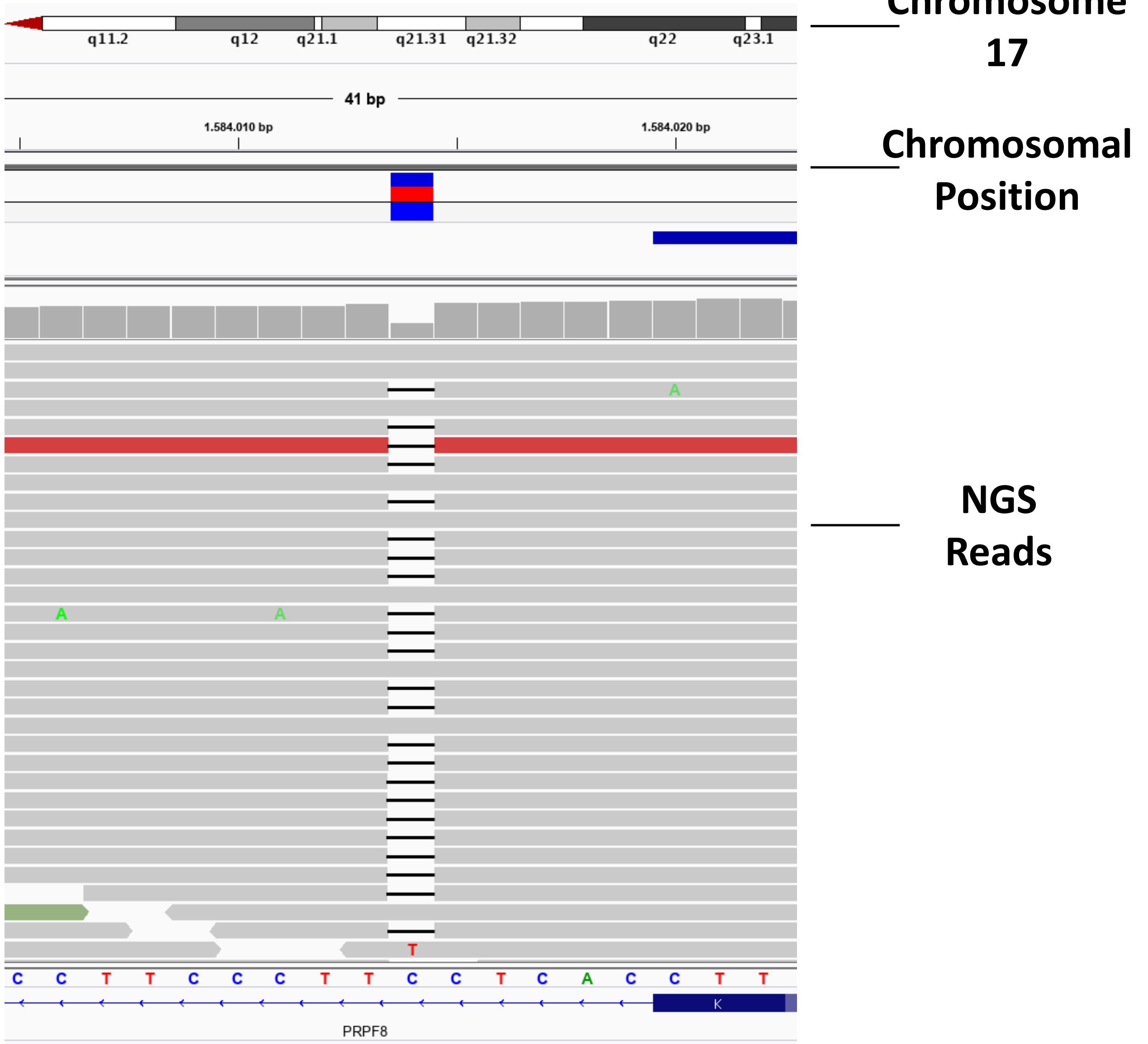

Patient 019

Disease-associated gene: CTNNA1

c.1310C>T (50x Total Count)

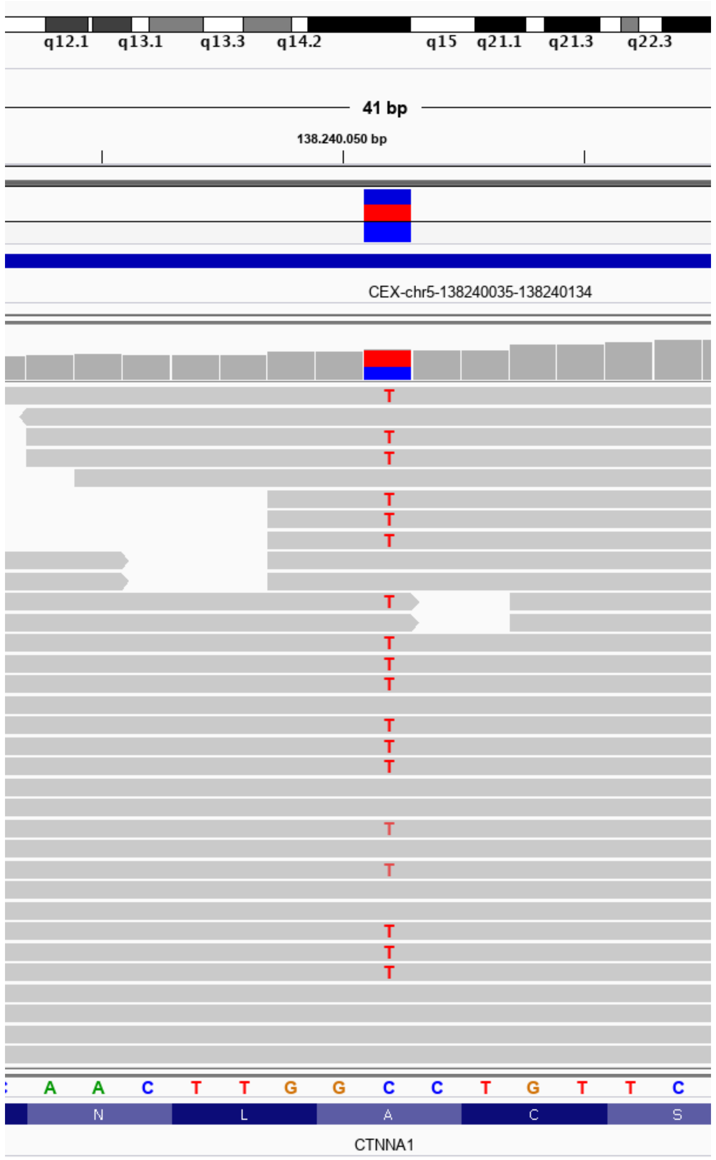

Chromosome 5

Chromosomal Position

NGS Reads

Patient 020

Disease-associated gene: OFD1

c.74A>G (256x Total Count)

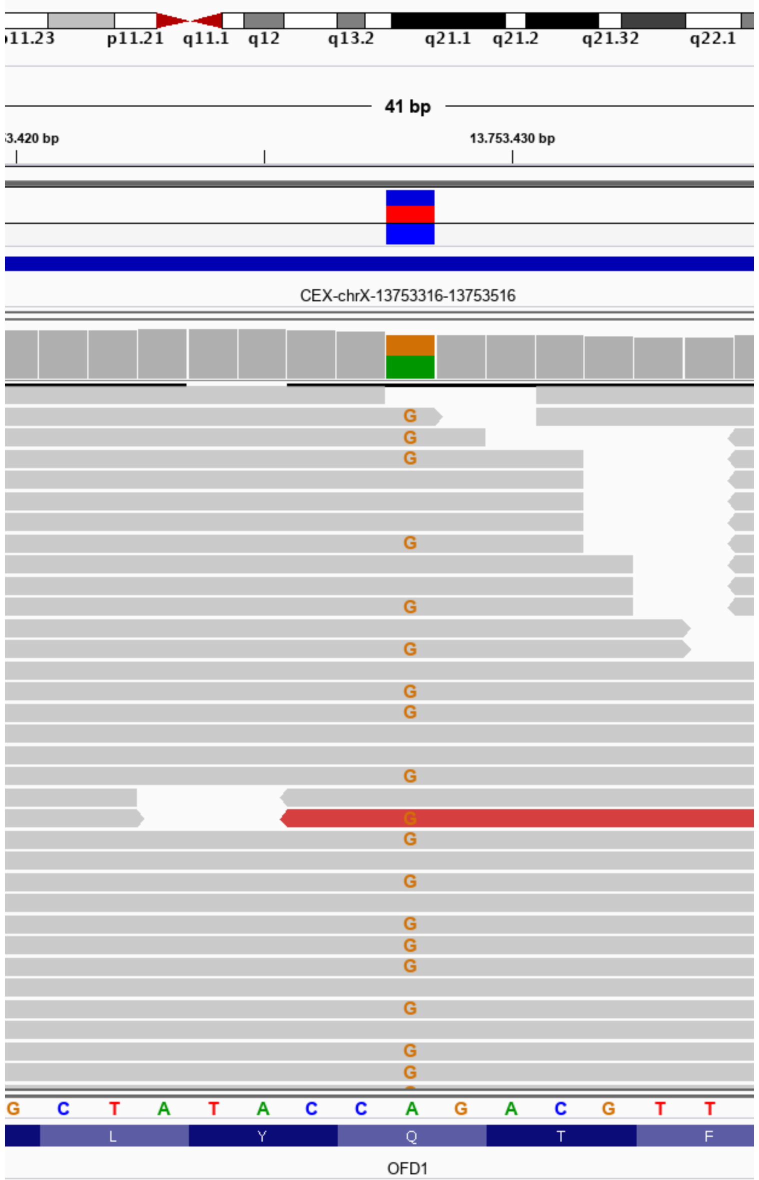

Chromosome X

Chromosomal Position

NGS Reads

Patient 022

Disease-associated gene: ABCA4

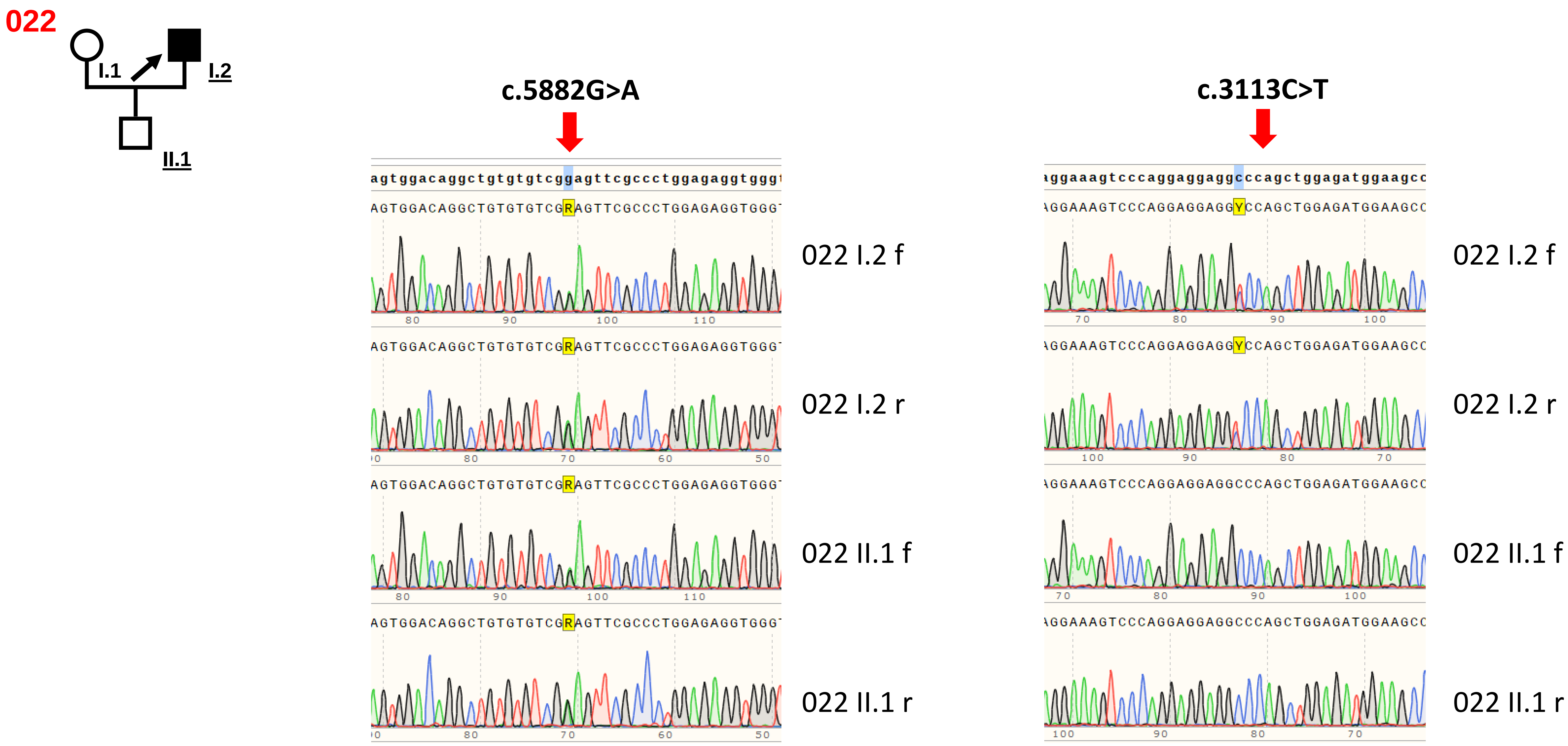

Patient 022

Disease-associated gene: ABCA4

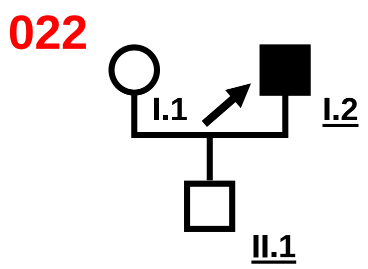

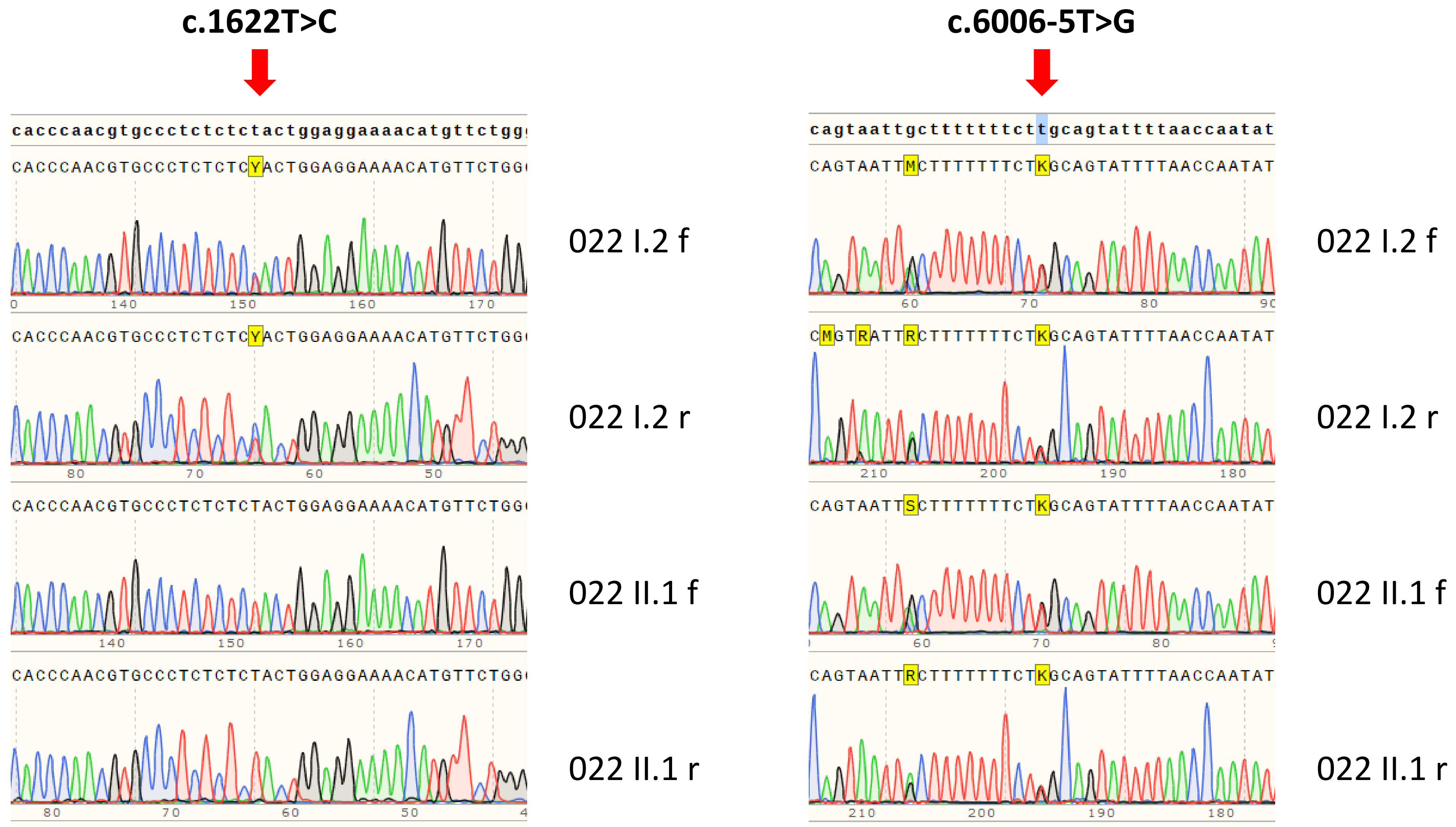

Patient 023

Disease-associated gene: ABCA4

c.4539+1770C>A

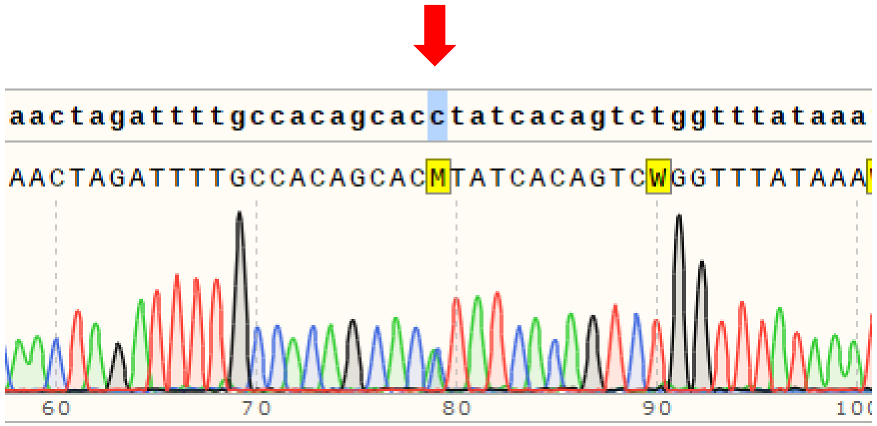

023 f

c.5196+1015A>G

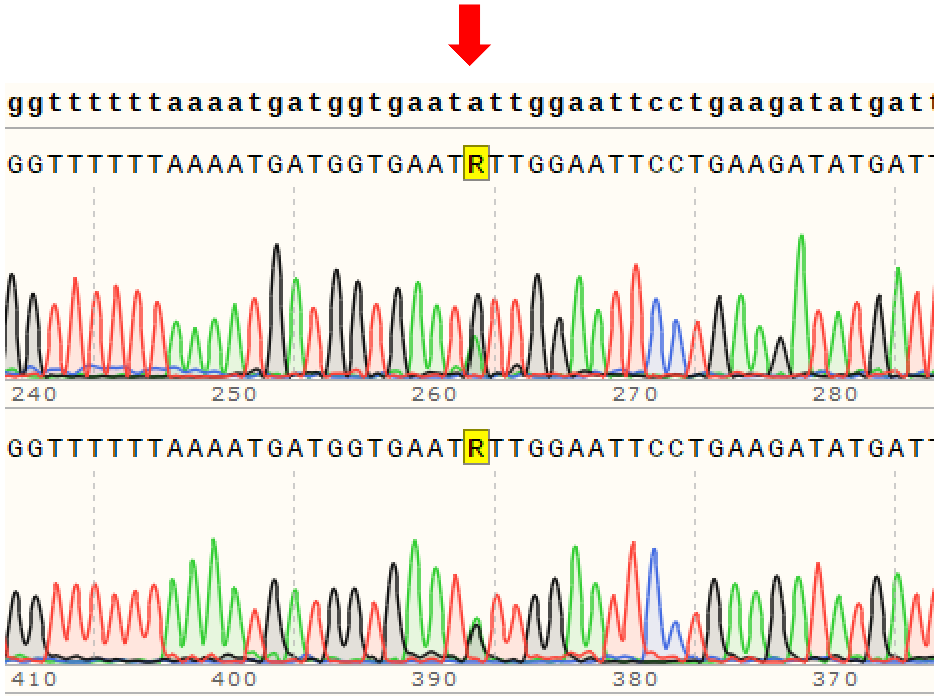

023 f

023 r

Patient 024

Disease-associated gene: ABCA4

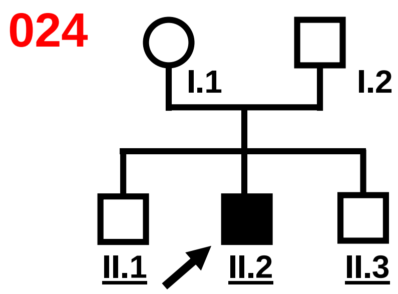

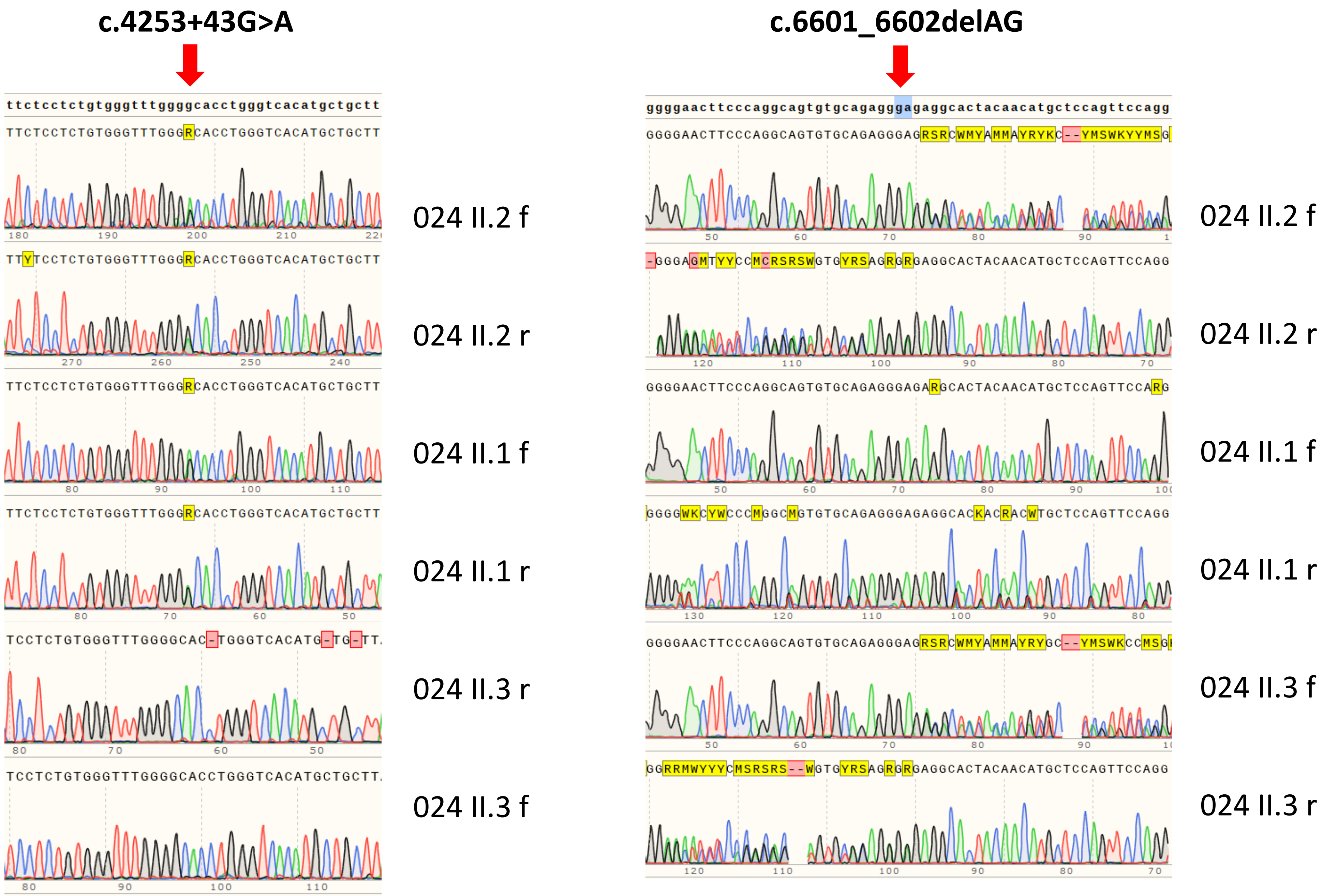

Patient 025

Disease-associated gene: ABCA4

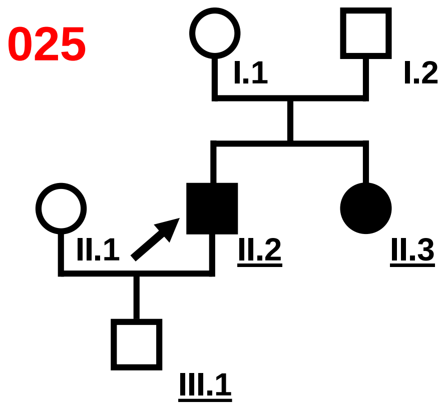

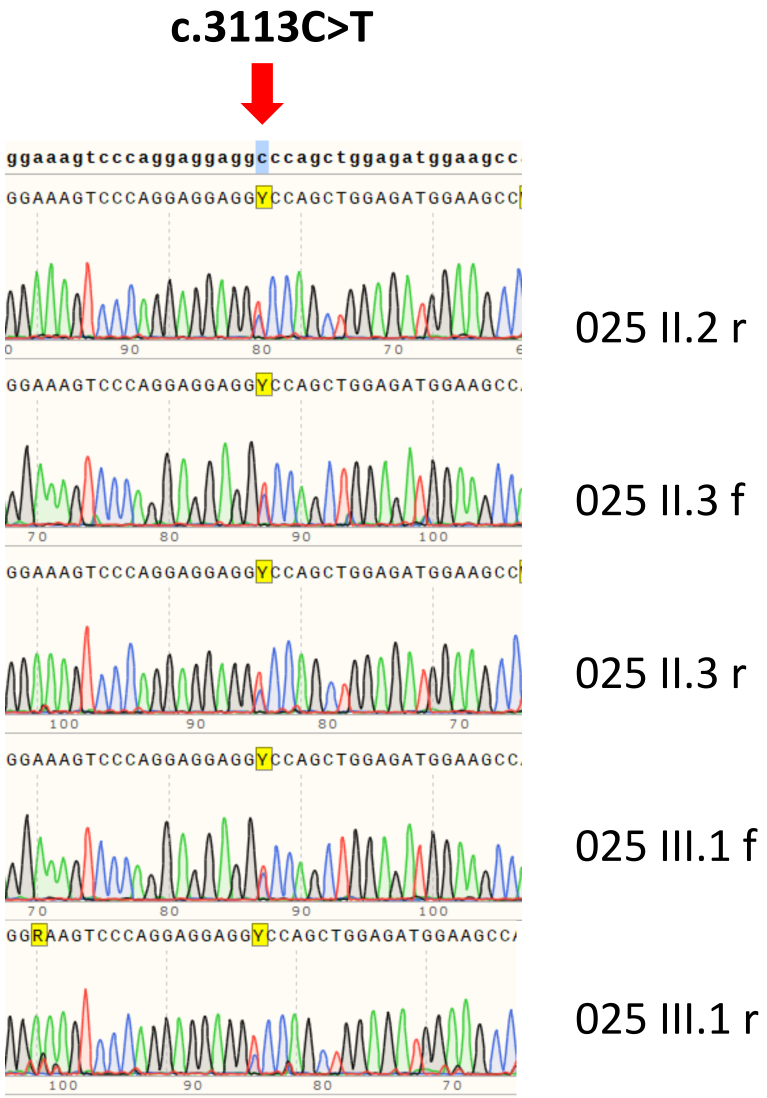

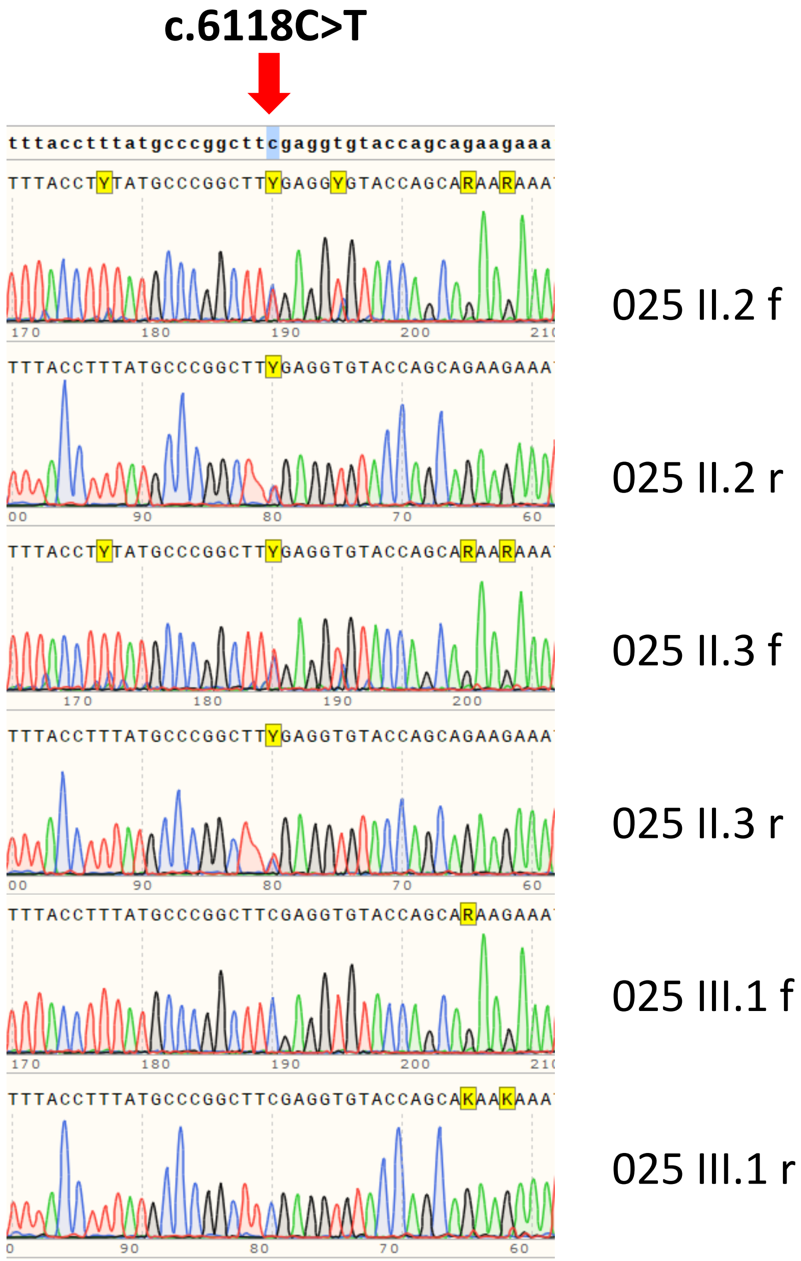

Supplement: Supplementary file 1 [file genes-12-01269-s001.zip › Supplementary Material Figure S1.pdf]
