# Supplementary material for: High-Throughput Sequencing to Identify Mutations Associated with Retinal Dystrophies
Source: Genes (Basel). 2021 Aug 20;12(8):1269. doi: 10.3390/genes12081269 (PMC8391535; doi:10.3390/genes12081269)
Supplement: Supplementary file 1 [file genes-12-01269-s001.zip › Supplementary Material Figure S2 .pdf]

Supplementary Materials

Figure S2. Fundus and OCT images of the patients in whom novel mutations were detected.

Patient 005

C2orf71
NM_001029883.2
c.[1709_1728del];[=], p.Gly570Glufs*3
c.[2655delT];[=], p.Ser885Serfs*2

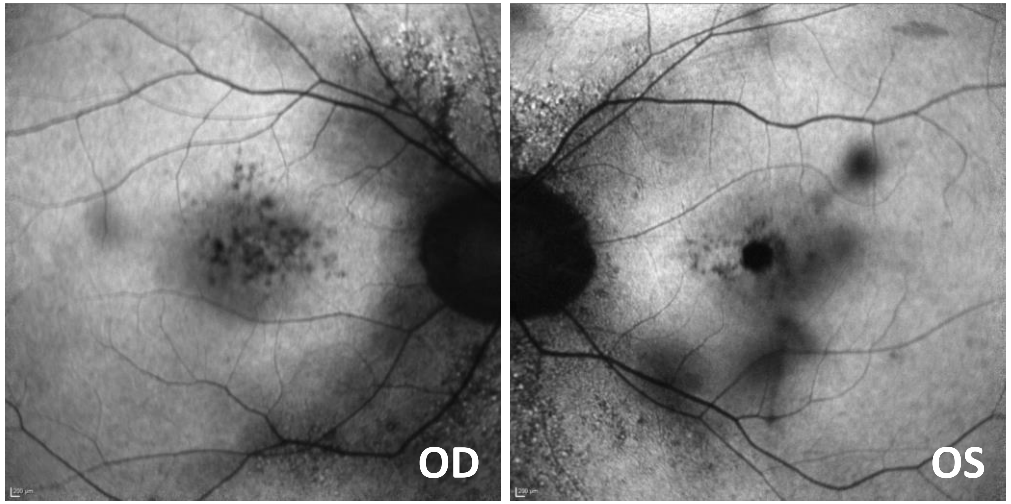

Fundus autofluorescence (FAF) images of patient 005. OD: oculus dexter, right eye. OS: oculus sinister, left eye.

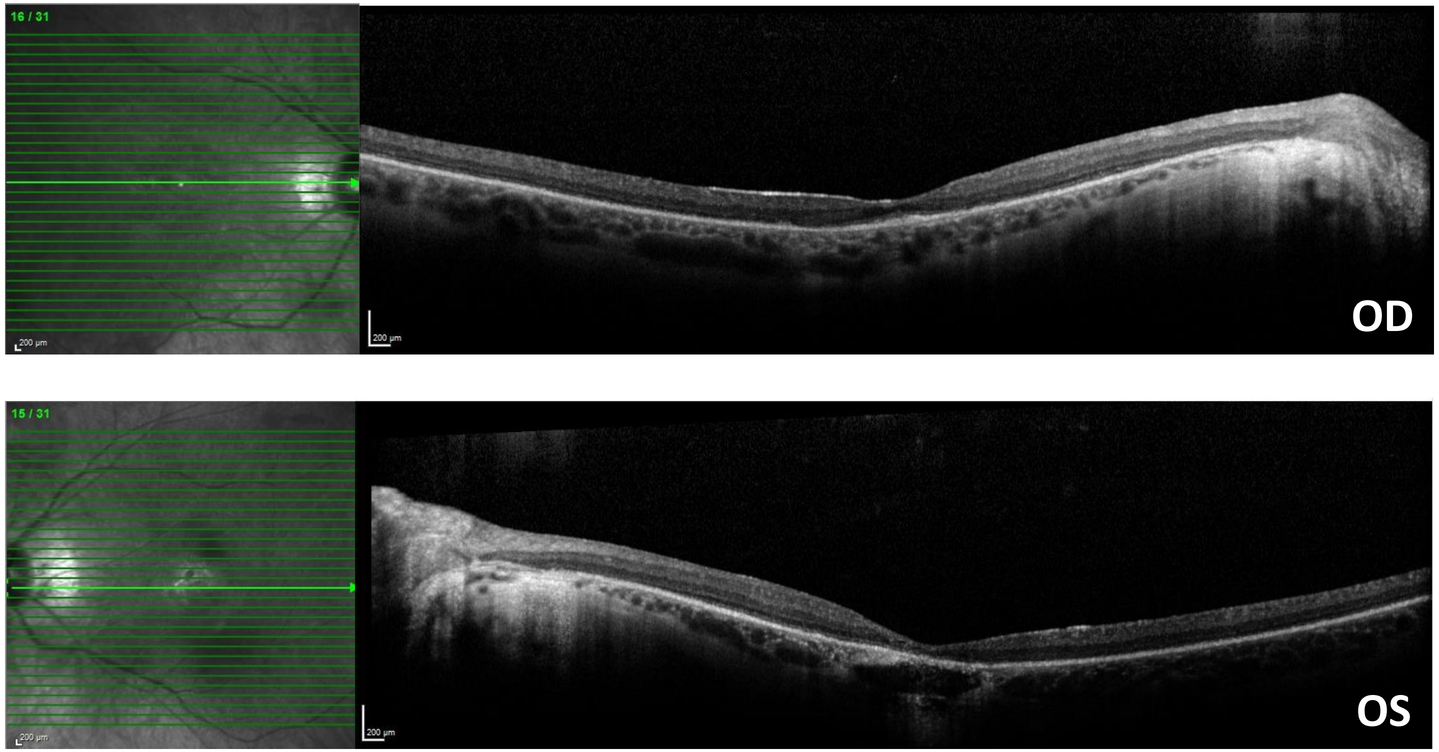

Optical coherence tomography (OCT) images of the macular region of patient 005. OD: oculus dexter, right eye. OS: oculus sinister, left eye.

Patient 008

RP1
NM_006269.1
c.[5957G>A];[=], p.Gly1986Asp

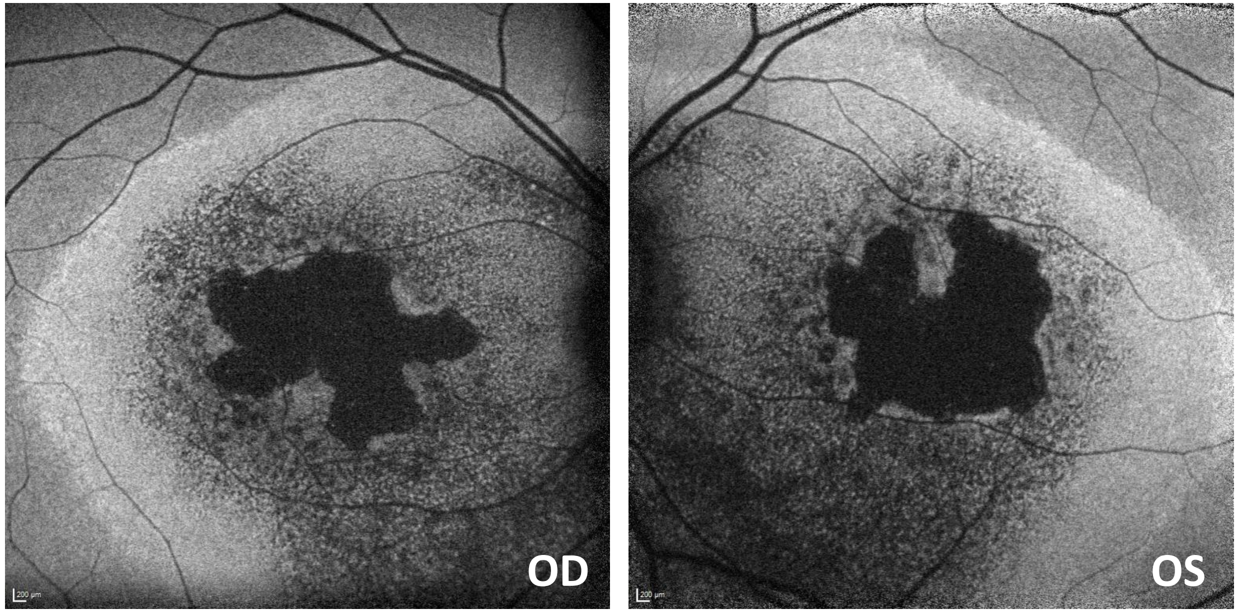

Fundus autofluorescence (FAF) images of patient 008. FAF images of both eyes show bilateral central hypoautofluorescent lesions with foveal sparing in the left eye, finely spreckled hypoautofluorescent spots and an adjacent hyperautofluorescent rim. OD: oculus dexter, right eye. OS: oculus sinister, left eye.

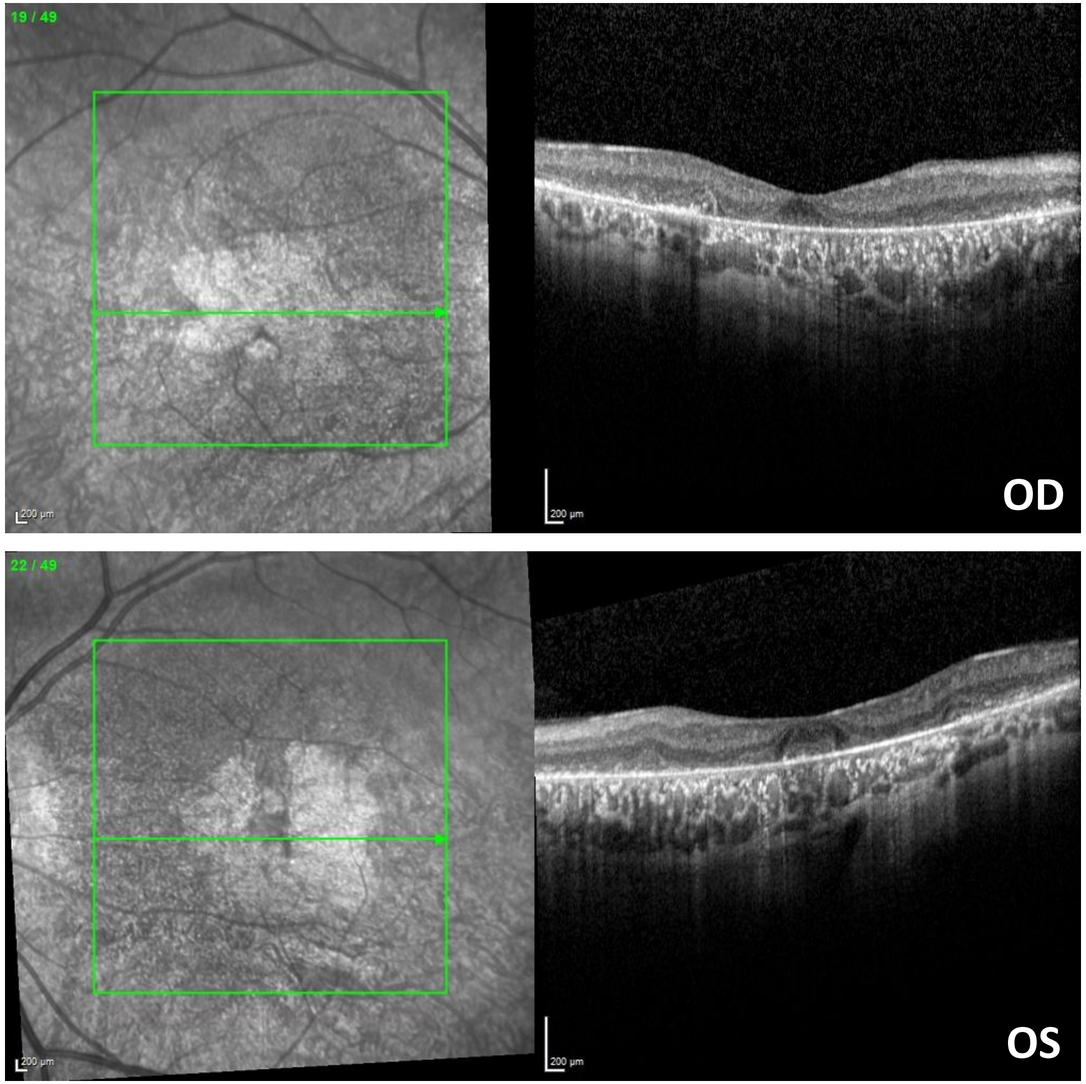

Optical coherence tomography (OCT) images of the macular region of patient 008. OCT images of both eyes show bilateral lesions of complete and incomplete retinal pigment epithelial and outer retinal atrophy; in the left eye, the fovea is excluded from complete atrophy. OD: oculus dexter, right eye. OS: oculus sinister, left eye.

Patient 010

RP1
NM_006269.1
c.[5957G>A];[=], p.Gly1986Asp

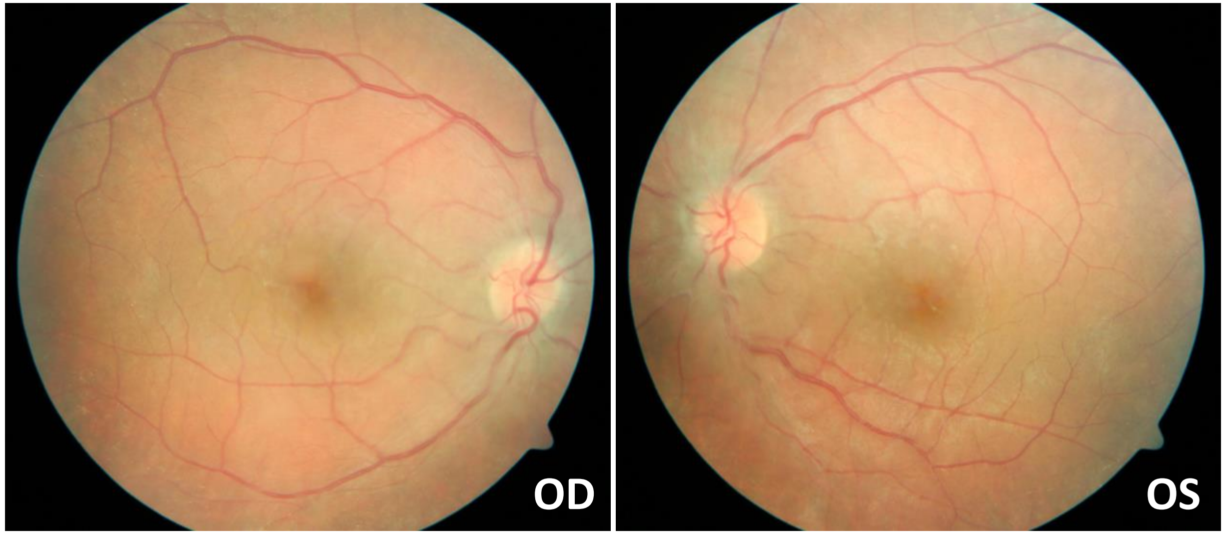

Fundus photography images of patient 010. Fundus photography images of the patient 010 show intraretinal cysts in the macular region. Right eye more pronounced than the left eye. OD: oculus dexter, right eye. OS: oculus sinister, left eye.

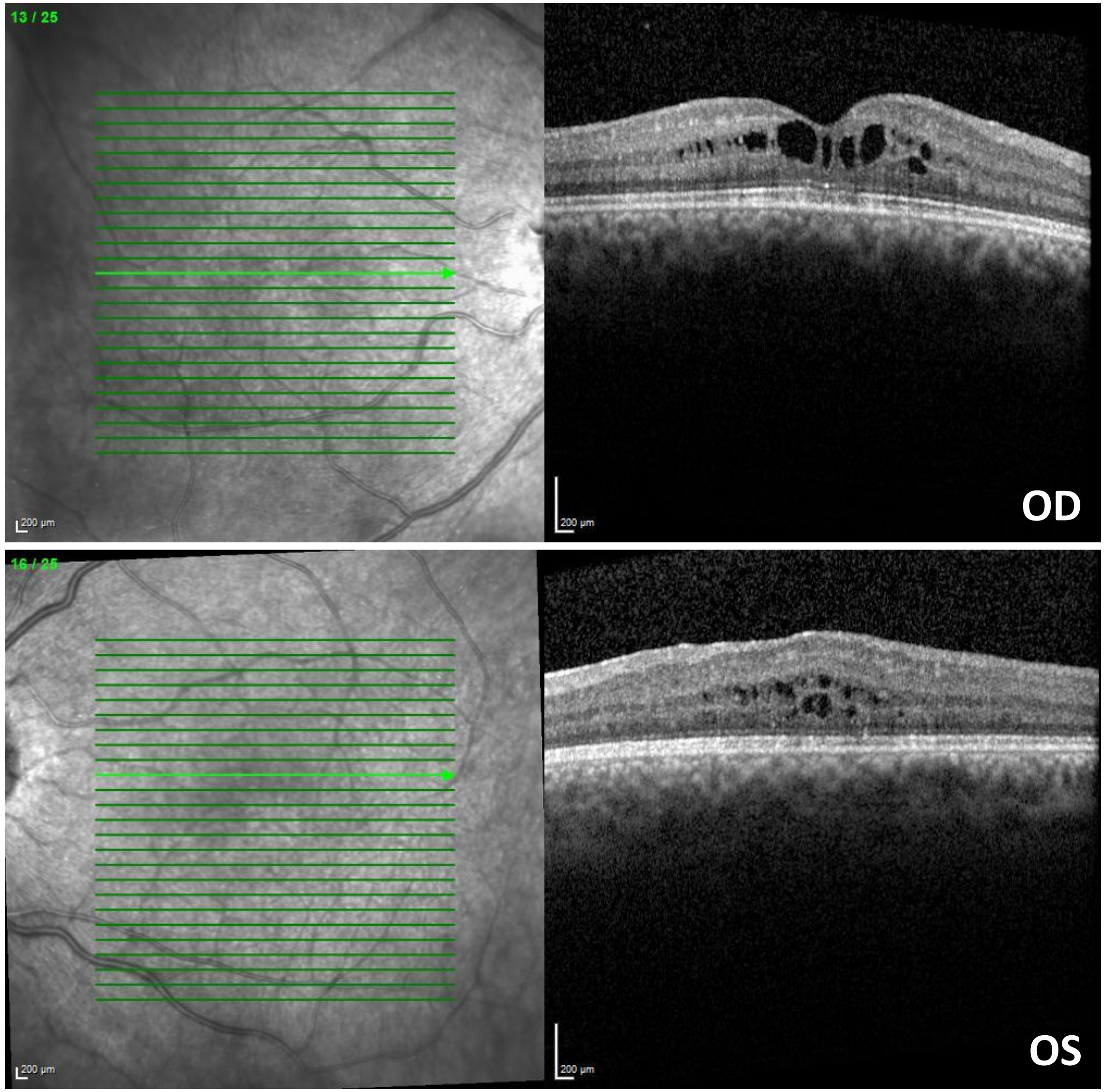

Optical coherence tomography (OCT) images of the macular region of patient 010. OCT images of the patient 010 show intraretinal cysts in the macular region. Right eye more pronounced than the left eye. OD: oculus dexter, right eye. OS: oculus sinister, left eye.

Patient 011

MAK
NM_001242957.3
c.[37G>A];[=], p.Gly13Ser
c.[1465+2dupT];[=], p.?

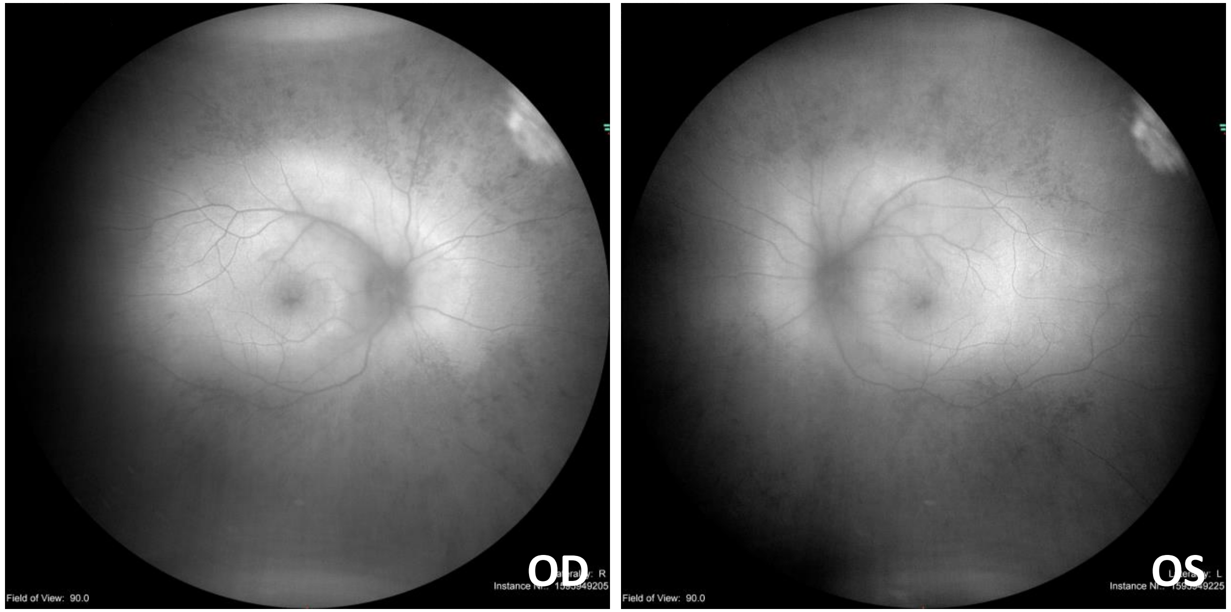

Fundus autofluorescence (FAF) images of patient 011. FAF images of both eyes show bilateral finely spreckled hypoautofluorescent spots in the midperiphery and periphery as well as paracentral hyperautofluorescence. OD: oculus dexter, right eye. OS: oculus sinister, left eye.

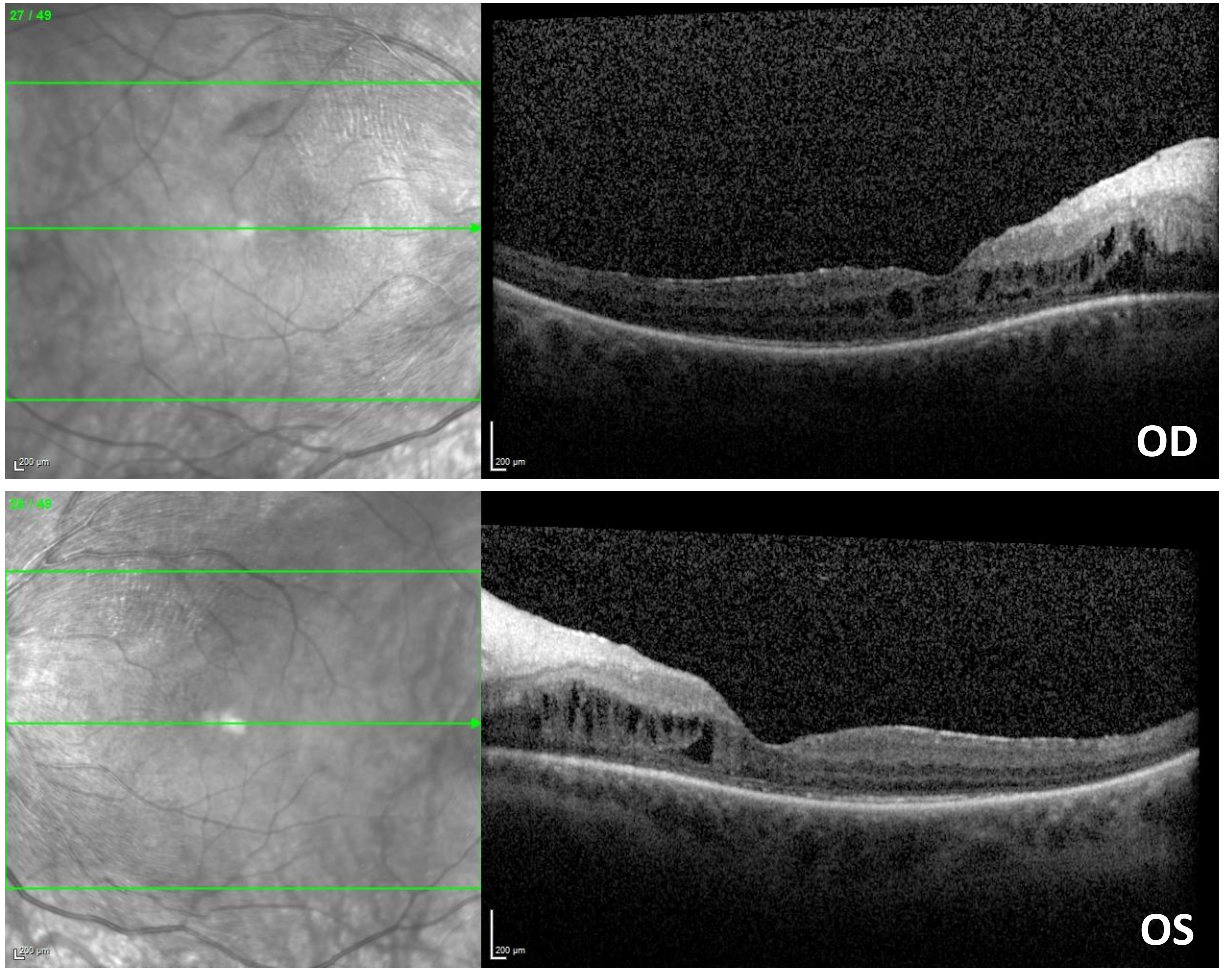

Optical coherence tomography (OCT) images of the macular region of patient 011. OCT images of both eyes show cystoid intraretinal spaces and incomplete atrophy of the photoreceptor layer starting two papilla diameter from the foveal centre. OD: oculus dexter, right eye. OS: oculus sinister, left eye.

Patient 014

PROM1
NM_006017
c.[1069G>C];[=], p.Val357Leu

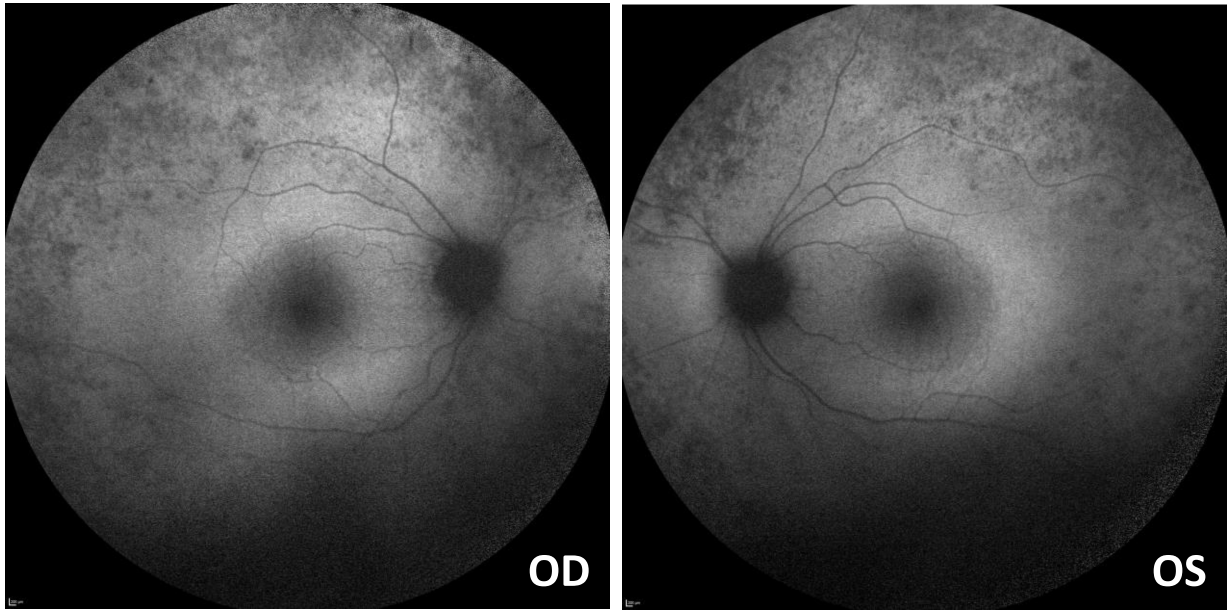

Fundus autofluorescence (FAF) images of patient 014. FAF images of both eyes show bilateral finely spreckled hypoautofluorescent spots in the midperiphery and periphery. OD: oculus dexter, right eye. OS: oculus sinister, left eye.

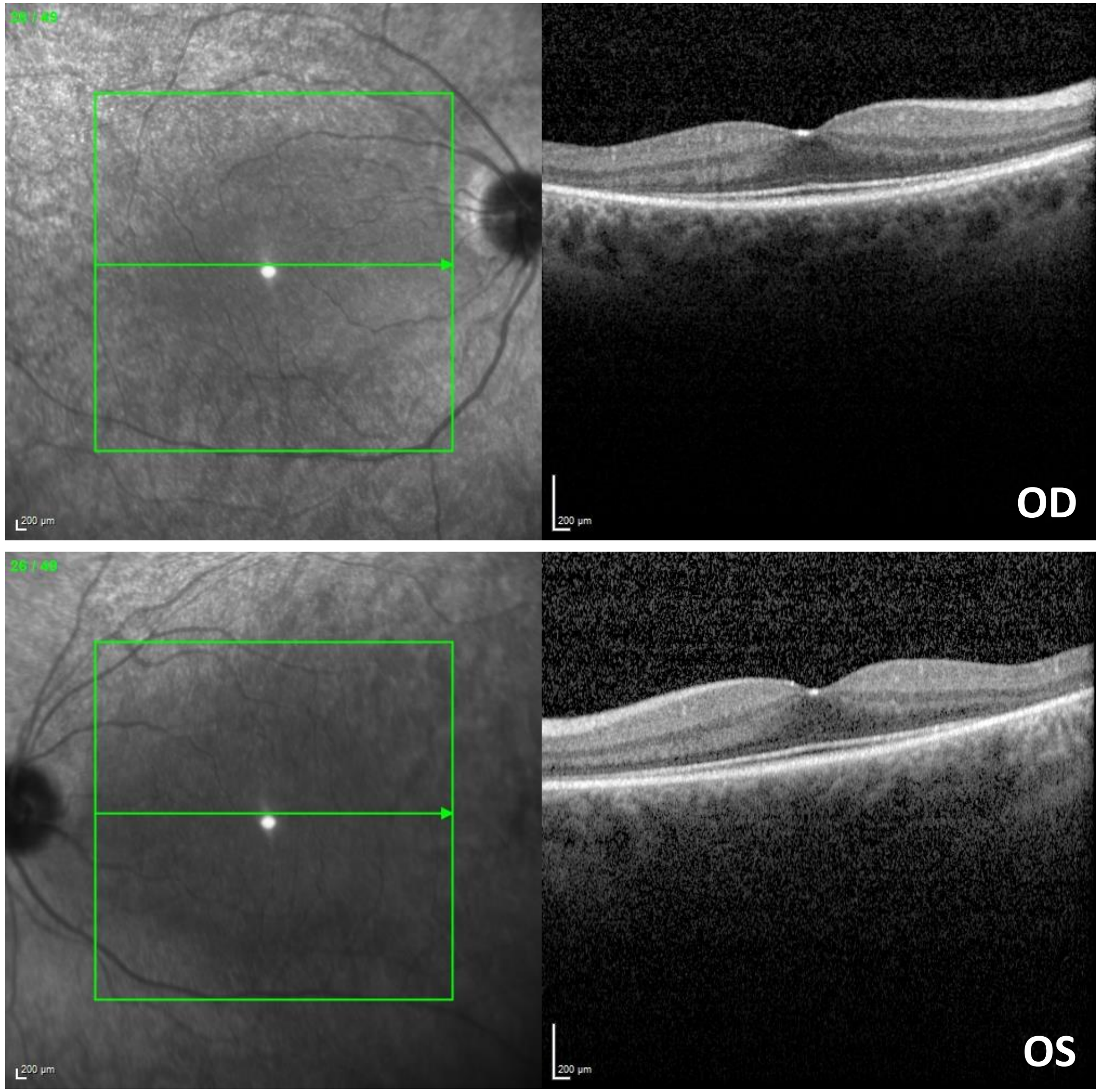

Optical coherence tomography (OCT) images of the macular region of patient 014. OCT images of both eyes show incomplete atrophy of the photoreceptor layer starting one papilla diameter from the foveal centre. OD: oculus dexter, right eye. OS: oculus sinister, left eye.

Patient 018

PRPF8,
NM_006445.3
c.[1098+6del];[=], p.?

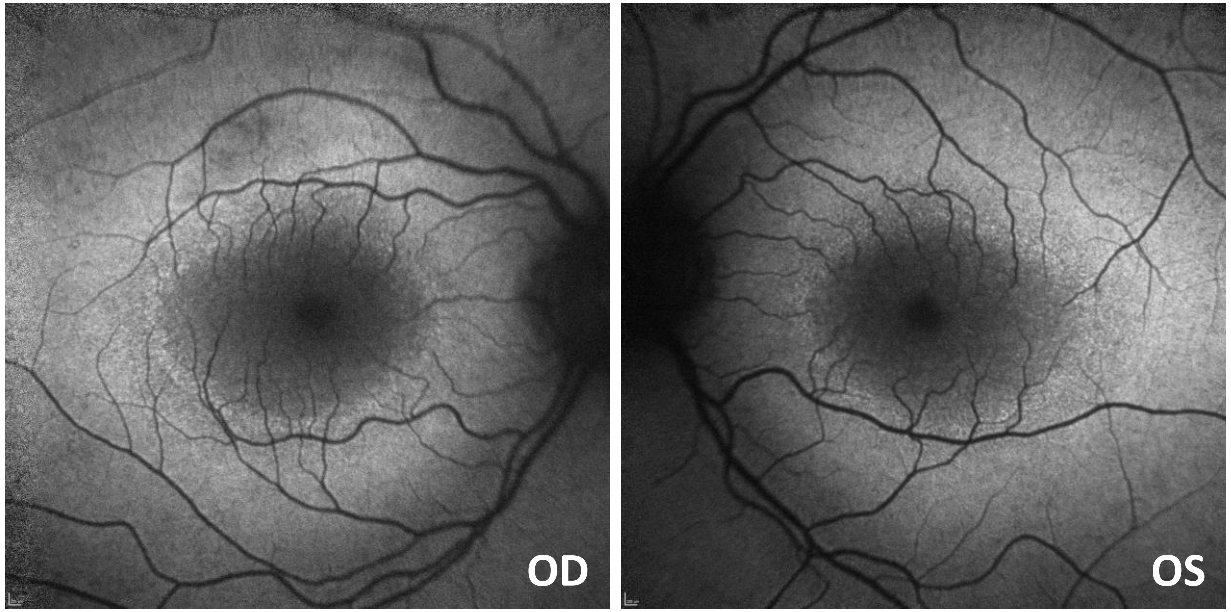

Fundus autofluorescence (FAF) images of patient 018. FAF images of both eyes show bilateral hypoautofluorescence in the central macula surrounded by a hyperautofluorescent rim. OD: oculus dexter, right eye. OS: oculus sinister, left eye.

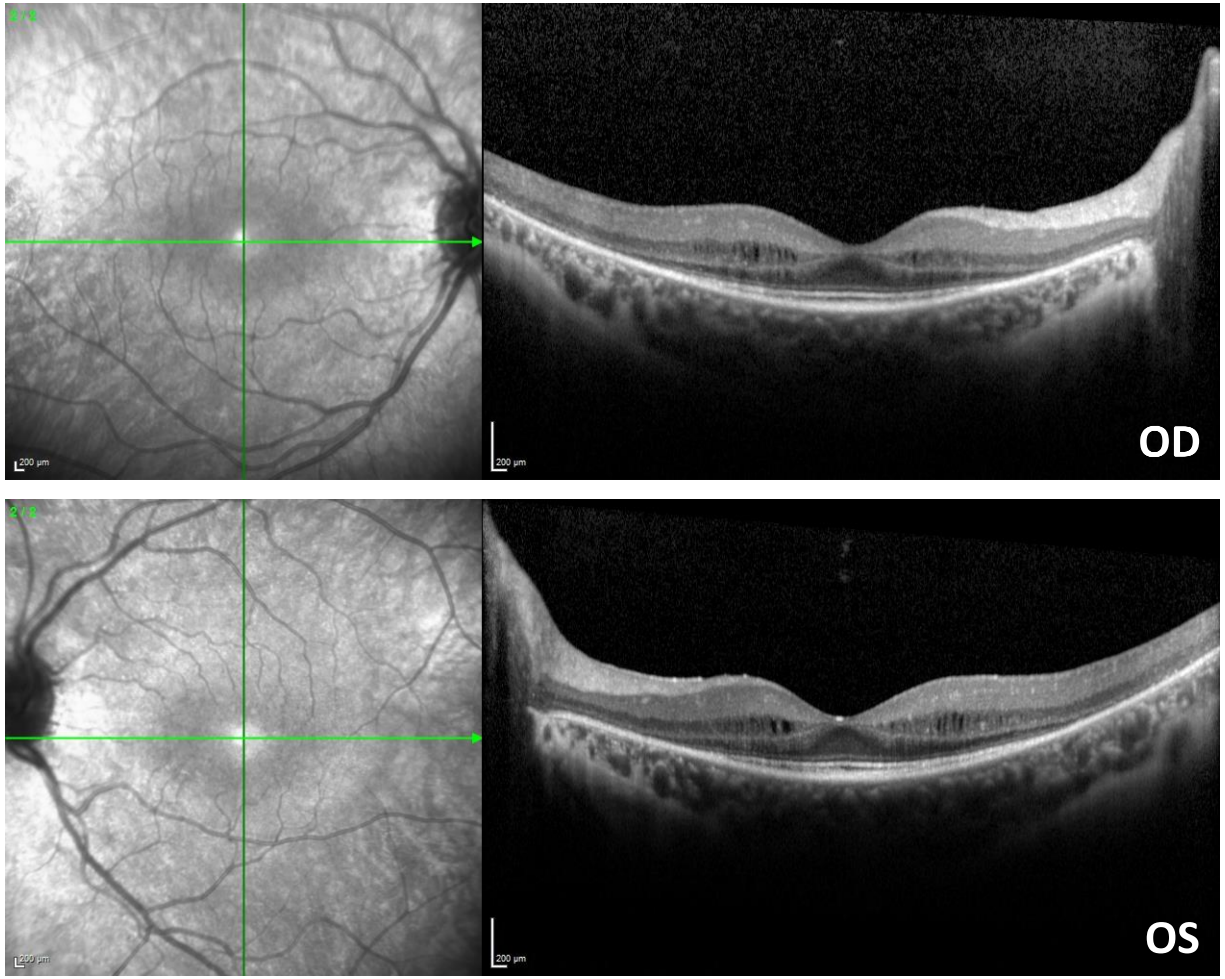

Optical coherence tomography (OCT) images of the macular region of patient 018. OCT images of both eyes show incomplete atrophy of the photoreceptor layer starting one papilla diameter from the foveal centre and cystoid intraretinal spaces mainly in the inner nuclear layer. The nerve fibre layer seems thickened.

Patient 020

OFD1
NM_003611.2
c.[74A>G];[=], p.Gln25Arg

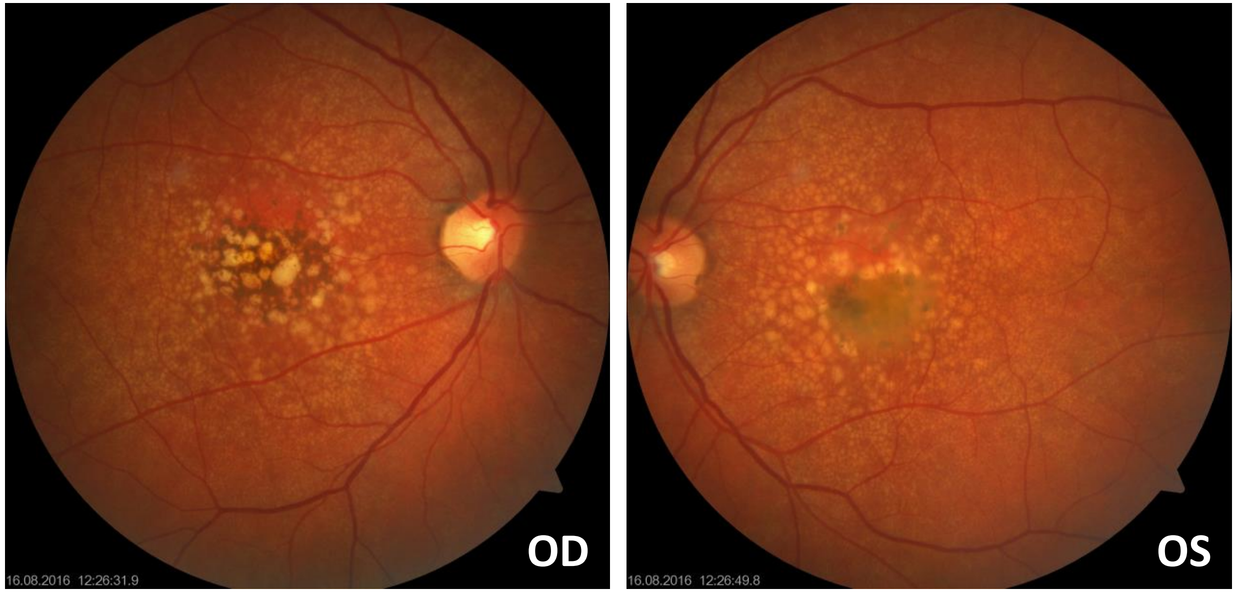

Fundus photography images of patient 020. Fundus photography images show numerous drusen especially centrally with dry neurosensory retina on the right eye. Left eye shows drusenoid pigment epithelial detachment. OD: oculus dexter, right eye. OS: oculus sinister, left eye.

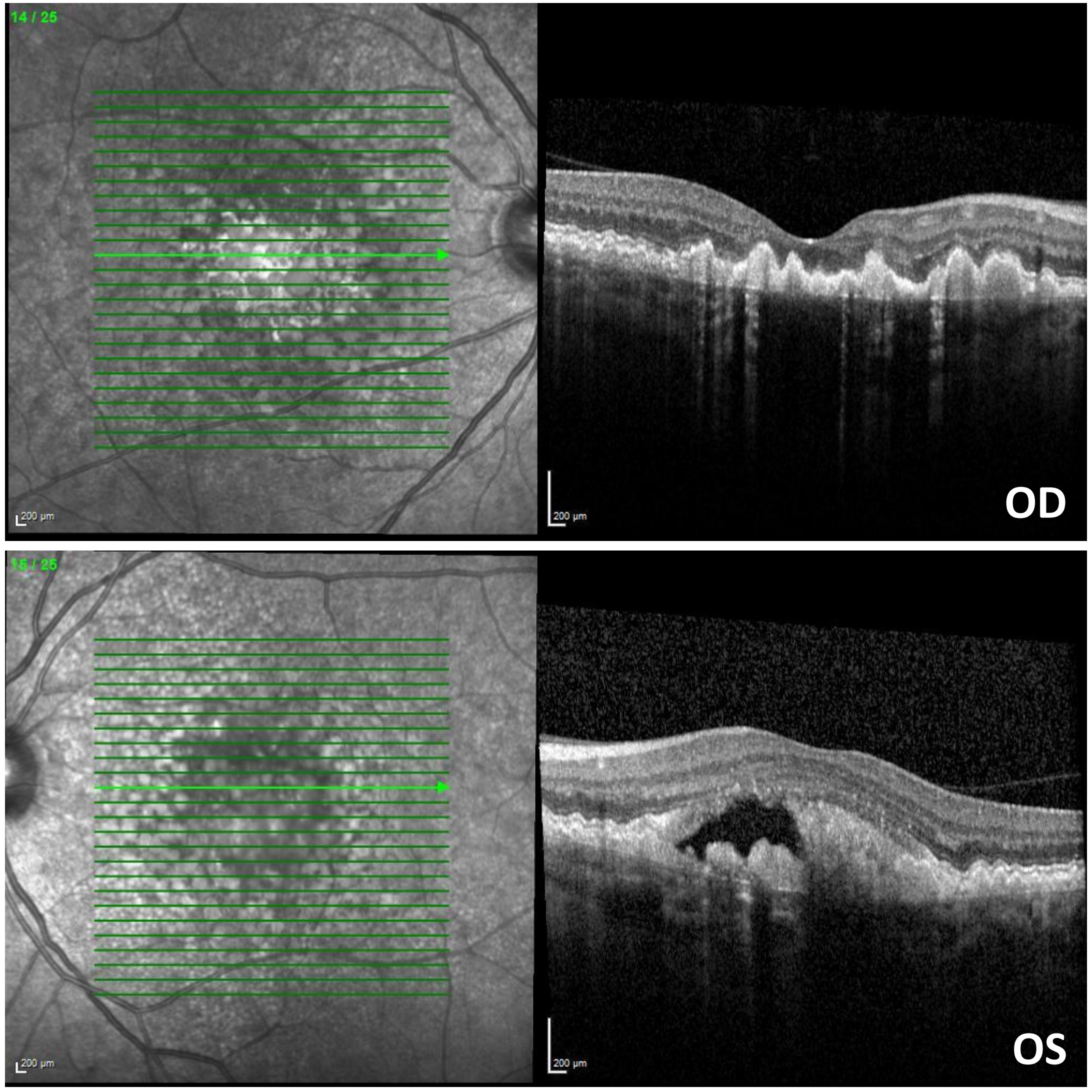

Optical coherence tomography (OCT) images of the macular region of patient 020. OCT images show numerous drusen especially centrally with dry neurosensory retina on the right eye. Left eye showing drusenoid pigment epithelial detachment. OD: oculus dexter, right eye. OS: oculus sinister, left eye.
