# Supplementary material for: High-Throughput Sequencing to Identify Mutations Associated with Retinal Dystrophies
Source: Genes (Basel). 2021 Aug 20;12(8):1269. doi: 10.3390/genes12081269 (PMC8391535; doi:10.3390/genes12081269)
Supplement: Supplementary file 1 [file genes-12-01269-s001.zip › Supplementary Materials Table S1.pdf]

Table S1. Clinical data of patients diagnosed with STGD or with the differential diagnosis of STGD.

Patient ID	Diagnosis	Age of onset	Severity	Visual acuity details	Best-corrected Visiual Acuity (LogMAR)		Tension	Fundus autofluorescence (FAF)	Autofluorescence and deposits (Severity)	optical coherence tomography (OCT)	central macular thickness	macular volume	Electroretinogram (ERG)	Fluorescein angiography	Color vision	Goldmann Visual	Others
					**OD**	**OS**											
008	CRD	45	severe	progressive visual impairment OU (blurry vision) over years, central vision distortion including metamorphopsia OU, night vision problems, reduced contrast sensitivity, glare, no color vision problems besides inherited protanopic color vision deficiency	0.1	0.1	16mmHg/16mmHg	bilateral finely spreckled hypoautofluorescent spots, adjacent hyperautofluorescent rim, (para-)central retinal atrophy, OS foveal sparing	deposits none, atrophy medium	bilateral (para-)central lesions cRORA, OD ORTs, OS foveal sparing, iRORA and retinal thinning up to the vascular arcades	177µm/231µm	6.21mm^3/6.40mm^3	ffERG:<br><br>scotopic and photopic amplitudes slightly reduced below normal values (combined rod cone dystrophy)	N/A	mainly protanopic confusions	OD>OS concentric scomoma	N/A
017	RP	childhood	severe	Initial photopsia, glare, night vision problems, reduced contrast sensitivity, variable visual impairment OU (blurry vision)	0.1	0.1	14 mmHg/15 mmHg	bilateral hypoautofluorescent spots, slight adjacent diffuse hyperautofluorescence	deposits none, atrophy medium (small lesions, great distribution)	bilateral indistinct borders of atrophy, mainly iRORA, foveal sparing, cystoid macular edema	286µm/270µm	7.55mm^3/7.31mm^3	ffERG:<br><br>reduced scotopic and photopic amplitudes, but near to normal	N/A	inconspicuous	regular borders of the visual field	N/A
019	STGD	20	N/A	Initial symptom: Central	0.8	1.2	N/A	OD: Mild perimacular	N/A	OD: Hyperreflective and irregular photoreceptor	205µm/221µm	8.42mm^3 / 8.89mm^3	N/A	OD: Mild perimacular staining, no	N/A	N/A	N/A

				Scotoma right eye in 2016				autofluorescence with flecks<br><br>OS: Inconspicuous		inner/outer segment junction<br><br>OS: Dry neurosensory retinal layers				leakage, no dark choroid<br><br>OS: Inconspicuous			
020	MD	51	N/A	No visual field defects in 2012	Date: Value<br><br>08/16: 0.05<br><br>05/16: 0.32<br><br>04/16: 0.4<br><br>02/16: 0.4<br><br>01/16: 0.5<br><br>12/15: 0.5<br><br>08/15: 0.5<br><br>05/15: 0.7<br><br>03/14: 0.8<br><br>03/11: 1.0	Date: Value<br><br>08/16: 0.3<br><br>05/16: 0.32<br><br>04/16: 0.5<br><br>02/16: 0.4<br><br>01/16: 0.5<br><br>12/15: 0.5<br><br>08/15: 0.63<br><br>05/15: 0.7<br><br>03/14: 0.8<br><br>03/11: 1.0	Date: Value<br><br>05/15: 18/19 mmHg<br><br>12/15: 20/20 mmHg<br><br>01/16: 22/22 mmHg<br><br>04/16: 16/16 mmHg	Central autofluorescence due to multiple Drusen (Drusen maculopathy)	N/A	OD: Numerous drusen especially centrally. Dry neurosensory retina<br><br>OS: Drusenoid pigment epithelial detachment	Date: Value<br><br>08/16: 170 µm/489 µm<br><br>06/15: 516 µm/504 µm	Date: Value<br><br>08/16: 8.63 mm^3 /10.65mm^3<br><br>06/15: 10.68 mm^3/10.69mm^3	N/A	OD: Intense staining, no leakage<br><br>OS: Intense staining, no leakage	N/A	N/A	Alopecia, Fibromyalgia.<br><br>Does not look like classical Stargardt but something like Drusenoid maculopathie
021	STGD	69	N/A	Left Eye: Eccentric vision, subjective visual impairment left eye when presenting in Nov. 2015	Hand movement	Finger-counting	N/A	Perifoveolar hyper-auto-fluorescence with flecks OS>OD, centrally no evaluation possible due to retinal pigment epithelial atrophy	N/A	Bilateral: Central pigment epithelial and central retinal atrophy	236µm/227µm	5.77 mm^3/6.23mm^3	N/A	OD>OS dark choroid, centrally large RPE defects. Late staining, no leakage	N/A	N/A	Developed Myelodysplastic syndrome 04/2017.<br><br>Arterial hypertension, Cardiac arrhythmias, post stent operation, fibromyalgia

022	STGD	30	N/A	N/A	2015: 1/35 (Visual acuity chart) 2011: 0.05	2015: 1/35 2011: 0.05	18mmHg/19mmHg	Bilateral: Diffuse paracentral and midperipheral autofluorescence, central large RPE defects	N/A	Bilateral: Central pigment epithelial and central retinal atrophy	132µm/70µm	4.39mm³/4.48mm³	N/A	Bilateral: Dark choroid Late-phase: Perimacular staining, no leakage	N/A	N/A	Hypertension, Diabetes II,
023	STGD	50	Mild	metamorphopsia OD>OS, glare, progressive visual impairment OU (blurry vision), reduced contrast sensitivity	0.22	0.22	12mmHg/12mmHg	bilateral alterations of the retinal pigment epithelium (RPE) and circumscribed areas of paracentral retinal atrophy (foveal sparing), surrounding hypo- and hyperautofluorescent drusenoid spots	deposits low, atrophy medium	circumscribed paracentral complete retinal pigment epithelium and outer retinal atrophy (cRORA), adjacent incomplete retinal pigment epithelium and outer retinal atrophy /iRORA), foveal sparing of RORA, degenerative intraretinal fluid	266µm/304µm	7.95mm³/8.08mm³	mfERG: OD>OS reduced amplitudes in the central area, some artefacts	N/A	inconspicuous (Panel D15)	regular borders of the visual field, OS (para-)central scotoma	N/A
024	STGD	50	Mild	progressive paracentral scotoma OU, progressive visual impairment OU (blurry vision), metamorphopsia	0.1	0.22	13mmHg/14mmHg	bilateral hyperautofluorescent macular and peripapillary spots, few spots of paracentral retinal atrophy (foveal sparing)	deposits medium, atrophy low	hyperreflective spots, partially invading into the outer retina, partly confluent lesions of cRORA, foveal sparing, degenerative intraretinal fluid	328µm/322µm	9.31mm³/9.00mm³	mfERG: bilateral amplitudes in normal range, slight relative reduction in the paracentral areas	FA: bilateral dark choroid, mild paracentral dye pooling in the late phase	Inconspicuous	N/A	Clinical examination: pattern-like distribution of the lesions
025	STGD	49	N/A	N/A	0.05	0.3	N/A	N/A	N/A	OD: Foveolar retinal thinning with intraretinal cysts and questionable retinal pigment epithelial atrophy	216µm/110µm	6.79mm³ / 6.99mm³	N/A	N/A	N/A	N/A	Decrease of VA at the age of 40. Sister has M. Stargardt (020 II.3)

										OS: Mild central retinal atrophy with reduced foveolar depression, signs of retinal pigment epithelial atrophy							
025II.3	STGD	23	N/A	N/A	Date: Value<br>02/87: 0.4<br>08/15: 0.05	Date: Value<br>02/87: 0.2<br>08/15: 0.05	Date: Value<br>02/87: 13/15 mmHg<br>08/15: 16/16 mmHg	OD: Central retinal pigment epithelial atrophy, perimacular hyperautofluorescent flecks, dark choroid<br>OS: Central retinal pigment epithelial atrophy, perimacular hyperautofluorescent flecks, dark choroid	N/A	OD/OS: Central retinal pigment epithelial and outer retinal atrophy	257µm / 192µm	7.12mm^3 / 6.96 mm^3	N/A	OD/OS: Central retinal pigment epithelial defect, macular hyperfluorescent flecks, late staining, no leakage	N/A	N/A	N/A
026	STGD	16	severe	Subjective visual impairment since 2013<br>visual field: Bilateral central scotoma in year 2016	Date: Value<br>3/16: 0.16<br>1/16: 0.2p	Date: Value<br>03/16: 0.2p<br>1/16: 0.2p	14 mmHg/14mmHg	OD/OS: Perimacular autofluorescent spots	N/A	OD/OS: Central foveal retinal pigment epithelial and retinal atrophy	20 µm / 61 µm	7.33mm^3 / 7.15 mm^3	OD/OS: Dark choroid, foveolar hyperfluorescence with central late staining, no leakage	N/A	N/A	Central scotoma	N/A

N/A: Not available.
